# Phospho-Site Mutations in Transcription Factor Suppressor of Hairless Impact Notch Signaling Activity During Hematopoiesis in *Drosophila*

**DOI:** 10.3389/fcell.2021.658820

**Published:** 2021-04-14

**Authors:** Lisa Frankenreiter, Bernd M. Gahr, Hannes Schmid, Mirjam Zimmermann, Sebastian Deichsel, Philipp Hoffmeister, Aleksandra Turkiewicz, Tilman Borggrefe, Franz Oswald, Anja C. Nagel

**Affiliations:** ^1^Department of General Genetics (190g), Institute of Biology (190), University of Hohenheim, Stuttgart, Germany; ^2^Department of Internal Medicine 1, Center for Internal Medicine, University Medical Center Ulm, Ulm, Germany; ^3^Institute of Biochemistry, Justus-Liebig University of Giessen, Giessen, Germany

**Keywords:** Notch signaling, Suppressor of Hairless, phosphorylation, hematopoiesis, *Drosophila*

## Abstract

The highly conserved Notch signaling pathway controls a multitude of developmental processes including hematopoiesis. Here, we provide evidence for a novel mechanism of tissue-specific Notch regulation involving phosphorylation of CSL transcription factors within the DNA-binding domain. Earlier we found that a phospho-mimetic mutation of the *Drosophila* CSL ortholog Suppressor of Hairless [Su(H)] at Ser^269^ impedes DNA-binding. By genome-engineering, we now introduced phospho-specific *Su(H)* mutants at the endogenous *Su(H)* locus, encoding either a phospho-deficient [*Su(H)^*S*269*A*^*] or a phospho-mimetic [*Su(H)^*S*269*D*^*] isoform. *Su(H)^*S*269*D*^* mutants were defective of Notch activity in all analyzed tissues, consistent with impaired DNA-binding. In contrast, the phospho-deficient *Su(H)^*S*269*A*^* mutant did not generally augment Notch activity, but rather specifically in several aspects of blood cell development. Unexpectedly, this process was independent of the corepressor *Hairless* acting otherwise as a general Notch antagonist in *Drosophila*. This finding is in agreement with a novel mode of Notch regulation by posttranslational modification of Su(H) in the context of hematopoiesis. Importantly, our studies of the mammalian CSL ortholog (RBPJ/CBF1) emphasize a potential conservation of this regulatory mechanism: phospho-mimetic RBPJ^*S*221*D*^ was dysfunctional in both the fly as well as two human cell culture models, whereas phospho-deficient RBPJ^*S*221*A*^ rather gained activity during fly hematopoiesis. Thus, dynamic phosphorylation of CSL-proteins within the DNA-binding domain provides a novel means to fine-tune Notch signal transduction in a context-dependent manner.

## Introduction

Seemingly simple signaling transduction mechanisms can induce a surprisingly large variety of cell types and organs and form the basis to understand regulatory networks governing development in higher organisms. As spatio-temporal aberrant signals or malfunctions are often linked to disease, including tumorigenesis, an understanding of the underlying processes is fundamental to therapeutic progress. A well-studied example is hematopoiesis, which occurs in two phases in mammals and *Drosophila* alike, to give rise to the various blood cell lineages (reviewed in: Kondo et al., 2003; Hartenstein, 2006; Letourneau et al., 2016; Banerjee et al., 2019). A small group of highly conserved signaling pathways and specific transcription factors govern hematopoiesis, allowing analyses in the *Drosophila* model system with broad implications for the understanding of mammalian hematopoiesis and leukemia development. For example, the Notch signaling pathway has been implicated in the regulation of hematopoiesis in mammals as well as in *Drosophila* (Allman et al., 2002; Evans et al., 2003; Radtke et al., 2005). In vertebrates, Notch is required for the generation of hematopoietic stem cells and also controls lymphoid cell fates, i.e., T-cell commitment (Bigas and Espinosa, 2012). The latter conceptually parallels the role of Notch in *Drosophila* hematopoiesis, where it plays an instructive role in the differentiation of crystal cells (Duvic et al., 2002). Crystal cells are the second most frequent blood cell type besides the predominant plasmatocytes that are phagocytic (Lebestky et al., 2000; Evans et al., 2003). Crystal cells play an important role in innate immunity and wound healing; by expressing prophenoloxidase they are able to induce melanization responses (Lebestky et al., 2000; Duvic et al., 2002). The development of crystal cells from hemocyte precursors depends on the AML-1 related transcription factor Lozenge (Lz). Notch signaling acts upstream of *lz* as well as in conjunction with Lz to promote crystal cell precursor maintenance, specification and differentiation (Lebestky et al., 2003; Terriente-Felix et al., 2013; Blanco-Obregon et al., 2020). Moreover, Notch activity is required continuously within the crystal cell to promote maturation, cell growth and survival (Mukherjee et al., 2011; Terriente-Felix et al., 2013; Miller et al., 2017).

The fundamental role of Notch is to mediate the intercellular communication amongst adjacent cells. At the first glance, the pathway appears rather simple: the signal sending cell presents a transmembrane ligand that is bound by the transmembrane Notch receptor in the neighboring signal-receiving cell. Upon binding of the ligand, the Notch receptor is cleaved to release the Notch intracellular domain (NICD). NICD then assembles a transcriptional activator complex together with the DNA-binding molecule CSL (CBF1/RBPJ in mammals, Suppressor of Hairless [Su(H)] in fly, and Lag-1 in worm) and the co-activator Mastermind, which drives the transcription of target genes (reviewed in: Borggrefe and Oswald, 2009; Bray, 2016; Kovall et al., 2017). In the absence of signal, CSL assembles repressor complexes on Notch target gene promoters involving different partners (Maier, 2006; Borggrefe and Oswald, 2009; Bray, 2016).

Repressors play an important role in tuning the appropriate Notch activity in a context-specific manner. In vertebrates several co-repressors directly interact with CSL, thereby competing with NICD for CSL binding and consequently inhibiting gene transcription (reviewed in: Borggrefe and Oswald, 2009; Borggrefe and Oswald, 2016). In *Drosophila*, the best characterized repressor is Hairless (H), which recruits further co-repressors Groucho (Gro) and C-terminal binding protein (CtBP) to silence Notch target gene expression (Bang and Posakony, 1992; Maier et al., 1992; Brou et al., 1994; Barolo et al., 2002; Nagel et al., 2005; Kurth et al., 2011). Structural data revealed that H not directly competes with NICD for binding to Su(H), but that instead a conformational change of the Su(H)-H complex precludes NICD binding (Maier et al., 2011; Yuan et al., 2016). Interestingly, genome-wide binding studies in cell culture and larval tissue demonstrated co-localization of Su(H) and H at many but not all Notch target loci, suggesting the existence of additional repressive mechanisms also in *Drosophila* (Chan et al., 2017).

Notch signaling involves inter-cellular and even inter-tissue communication (Boukhatmi et al., 2020). Accordingly, context-specific crosstalk with other signaling cascades and tissue specific modifications of Notch pathway members are well established (reviewed in: Andersson et al., 2011; Bray, 2016; Ho and Artavanis-Tsakonas, 2016; Kovall et al., 2017; Zhang et al., 2018). A prominent target of posttranslational modulation is the Notch receptor itself: Glycosylation of the extracellular domain of Notch affects the efficacy of Notch receptor activation by its ligands, and ensures a specific and robust activation of Notch and its downstream targets along the dorso-ventral (d/v) boundary of the wing precursors (reviewed in: Fortini, 2009). In addition, the Notch receptor is targeted by several kinases, affecting activator complex formation and stability that consequently either positively or negatively influence Notch signaling readout, depending on the mediating kinase and targeted region of NICD (reviewed in: Lee et al., 2015; Carrieri and Dale, 2017). Moreover, further downstream members were described as kinase targets, thereby influencing their performance in the pathway: intersections occur, e.g., between EGFR-activated MAPK and the downstream effector E(spl)m8 during eye development to coordinate photoreceptor development in a timely manner (Bandyopadhay et al., 2016). Furthermore, Gro and Su(H) are two other well defined substrates, whose activity is mitigated by MAPK-dependent phosphorylation in various N-dependent processes (Hasson et al., 2005; Auer et al., 2015). Recently, we identified a further phosphorylation to occur on Serine 269 (S269) in Su(H) isolated from *Drosophila* Schneider S2 cells, may impact Notch signaling by perturbing DNA-binding of Su(H) (Nagel et al., 2017). As this might influence both, N-dependent activation and repression, a careful spatio-temporal investigation of N-dependent processes regarding this modification is needed. Moreover, as the earlier work was based on misexpression experiments, the biological role of Su(H) phosphorylation at S269 remains unknown. To address these issues, we replaced the native *Su(H)* locus with a phospho-deficient [*Su(H)^*S*269*A*^*] or a phospho-mimetic [*Su(H)^*S*269*D*^*] isoform by genome engineering (Huang et al., 2009; Praxenthaler et al., 2017) and measured Notch activity in different N-dependent settings. As expected for *Su(H)* alleles defective in DNA-binding, *Su(H)^*S*269*D*^* mutants were homozygous lethal and displayed phenotypes of perturbed Notch activity in every context tested. In contrast, the phospho-deficient *Su(H)^*S*269*A*^* mutant not generally augmented Notch activity but showed a gain in Notch activity specifically during blood cell development. Moreover, we found no regulatory input by the general antagonist Hairless in this context. Hence, we postulate that phosphorylation of Su(H) is a novel mechanism to modulate Notch signaling activity in a tissue-specific manner, notably during hematopoiesis. As the phospho-target site is conserved in the mammalian CSL orthologs, it might be used there for crosstalk as well. Our murinized fly model and human cell systems underscore this assumption.

## Results

### Generation of Phospho-Site Specific *Su(H)* Mutants in the Endogenous Locus

**FIGURE 1:**
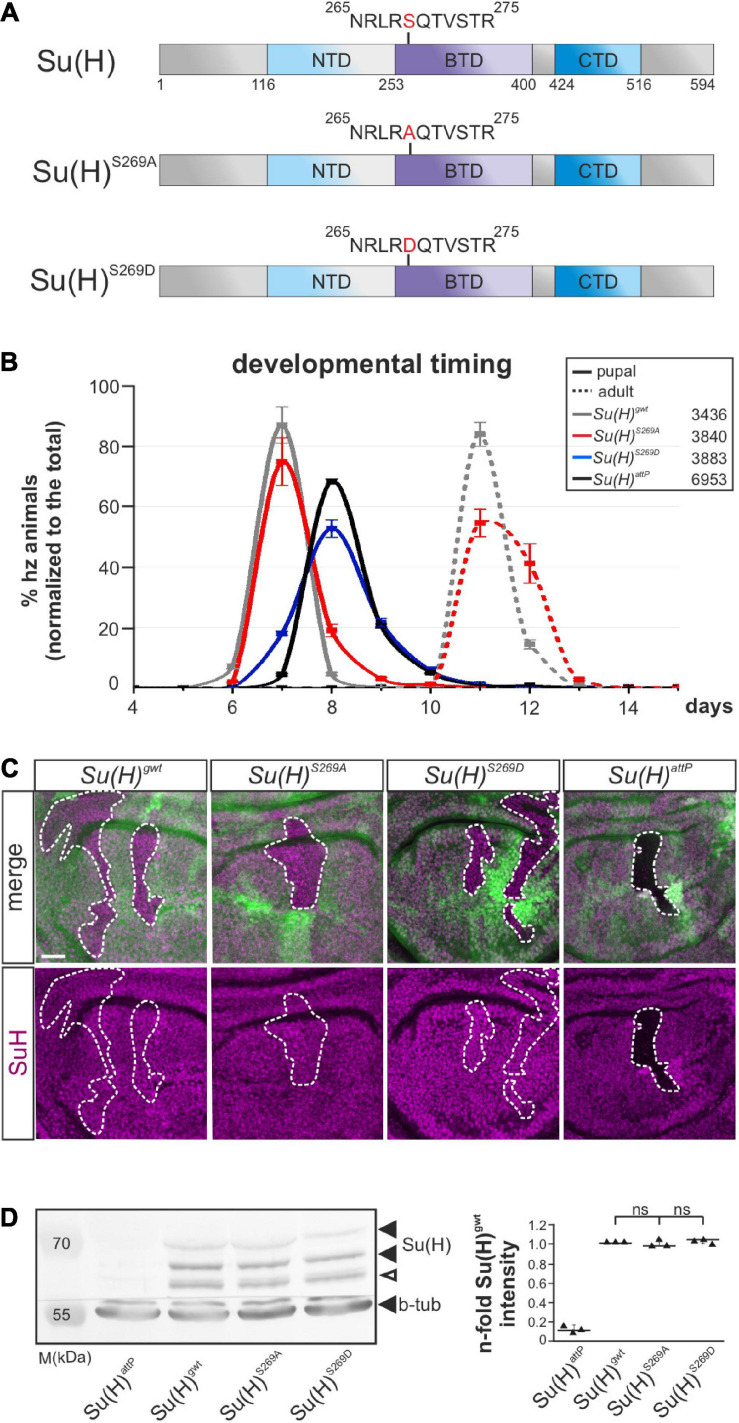
Developmental timing and protein analyses of specific *Su(H)^*S*269^* phospho-mutants. **(A)** Two phospho-specific *Su(H)* mutants were generated by genome engineering: *Su(H)^*S*269*A*^* (phospho-deficient) and *Su(H)^*S*269*D*^* (phospho-mimetic), replacing serine 269 with either alanine (S269A) or aspartic acid (S269D). Shown are the schemes of the Su(H)^*wt*^, Su(H)^*S*269*A*^, and Su(H)^*S*269*D*^ proteins, including the three domains, N-terminal (NTD, light blue), beta-trefoil (BTD, purple), and C-terminal domain (CTD, dark blue). **(B)** Pupal formation (solid lines) and eclosion of adults (broken lines), respectively, monitored over time (in days after egg deposition), is given as percentage of homozygotes normalized to the total number of offspring. Ten experiments were sampled; s.d. is depicted. Genotypes and total number of animals analyzed are given in the box. **(C)** Cell clones were induced in wing imaginal disks to monitor Su(H) protein abundance (magenta): wild type cells are marked with GFP, homozygous cells of the indicated genotypes lack GFP (encircled by a dashed line). No difference in protein abundance and localization could be detected between the *Su(H)* phospho-mutant variants and *Su(H)^*gwt*^*, whereas *Su(H)^*attP*^* null mutant clones were devoid of Su(H) protein as expected. Scale bar represents 25 μm for all panels. **(D)** Left panel: Western blot of protein extracts from homozygous larvae of the given genotype. Su(H) protein levels were not reduced in the Su(H) phospho-mutants (black arrowheads); the lower band presumably reflects a degradation product (open arrowhead). Beta-tubulin (b-tub) was used as loading control within the same blot cut before probing. M, prestained protein marker; approximate sizes are given in kDa. Right panel: Signal quantification of *n* = 3 independent blots. Data are mean ± s.d. with individual data points indicated. No significant difference between the phospho-mutants and wild type was observed (ns, not significant; *p* > 0.05).

Previously, we showed that Su(H) is phosphorylated at Serine 269 in *Drosophila* S2 cells, and that the introduction of a negative charge at this position interfered with the DNA-binding capability and transcriptional activity of Su(H). In addition, tissue-specific overexpression studies suggested that both, activation and repression of Notch signaling activity might be regulated by the phosphorylation of Su(H) at this site (Nagel et al., 2017). Based on the dominant negative character of the overexpression phenotypes, we were unable to decide on the biological relevance of the phosphorylation *in vivo*. To elucidate the implications of this posttranslational modification in the fly, we aimed to generate respective *Su(H)* mutants by genome engineering (Huang et al., 2009; Praxenthaler et al., 2017). To this end, the native *Su(H)* locus was replaced with constructs encoding the phospho-specific Su(H) replacement mutants *Su(H)^*S*269*A*^* and *Su(H)^*S*269*D*^*, representing a phospho-deficient and phospho-mimetic *Su(H)* variant, respectively (Figure 1A).

*Su(H)^*S*269*D*^* mutants were developmentally delayed and died before pupation, comparable to what was observed for the null allele *Su(H)^*attP*^*, and in agreement with a functional loss due to impaired DNA-binding (Figure 1B) (Nagel et al., 2017; Praxenthaler et al., 2017). By contrast, the phospho-deficient *Su(H)^*S*269*A*^* mutants were nearly indistinguishable from the *Su(H)^*gwt*^* wild type control with respect to developmental timing and hatching rate (Figure 1B). Moreover, both Su(H)^*S*269^-mutant proteins were indistinguishable from wild type protein in a clonal analysis regarding the subcellular distribution and amount (Figure 1C). The latter was confirmed in a quantification of the Su(H) proteins from larval extracts (Figure 1D), demonstrating similar expression and stability of the mutant proteins. Thus, the *Su(H)* phospho-site mutants do not bias Su(H) protein stability or localization, but Su(H) pseudo-phosphorylation compromises the survival of the fly.

### Impact of *Su(H)* Phospho-Site Mutants on Notch Signaling Activity During Embryonic and Larval Development

**FIGURE 2:**
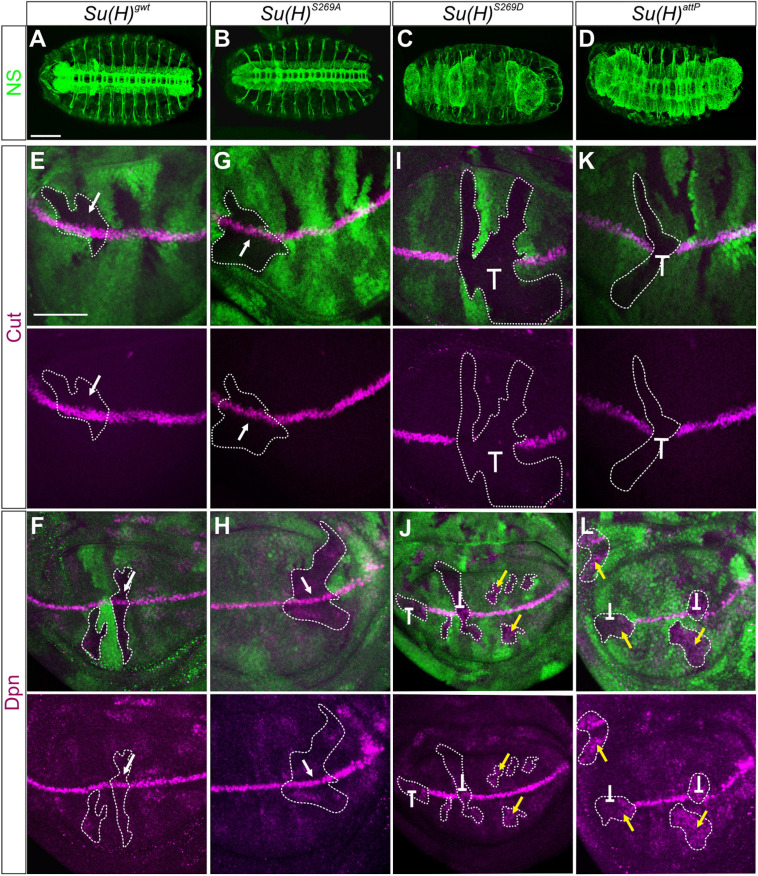
Clonal analysis during embryonic and larval development. **(A–D)** Nervous system (NS) stained with anti-HRP (green) revealed a wild type pattern in homozygous *Su(H)^*gwt*^* **(A)** and *Su(H)^*S*269*A*^* mutant embryos **(B)**, whereas neural hyperplasia was observed in *Su(H)^*S*269*D*^* and *Su(H)^*attP*^* germline clones **(C,D)**. Scale bar represents 100 μm. **(E–L)** Homozygous cell clones were generated in wing disks of *Su(H)^*gwt*^* **(E,F)**, *Su(H)^*S*269*A*^* **(G,H)**, *Su(H)^*S*269*D*^* **(I,J)**, and *Su(H)^*attP*^* **(K,L)** heterozygous animals. Expression of the Notch targets Cut (**E,G,I,K**, magenta) and Dpn (**F,H,J,L**, magenta) is shown. Homozygous mutant clones are marked by the absence of GFP (green; examples are outlined). In *Su(H)^*gwt*^* and *Su(H)^*S*269*A*^* clones, target gene expression is unaffected (**E–H**, arrows). In contrast, *Su(H)^*S*269*D*^* and *Su(H)^*attP*^* mutant clones show a loss of Cut and Dpn expression at the d/v boundary (**I–L**, repressive bar), whereas Dpn is de-repressed in mutant clones outside this region (**J,L**, yellow arrows). Scale bar in panel **(E)** represents 50 μm for all panels.

To determine the biological consequences of the phospho-site specific *Su(H)* mutants during development, functional analyses were performed aiming at following Notch signaling activity in different developmental settings. During embryonic neurogenesis Notch signaling activity restricts the number of neuroblasts by lateral inhibition. Accordingly, *N* mutant embryos display an excess of neurons at the expense of hypoderm (Simpson, 1990). *Su(H)* mutant embryos show this neurogenic phenotype only when lacking the maternal complement (Lecourtois and Schweisguth, 1995). Therefore, the dominant female sterile technique was applied to generate embryos completely devoid of wild type *Su(H)* protein (Chou and Perrimon, 1992). Anti-horseradish peroxidase (HRP) staining of neurons (Wang et al., 1994) revealed a wild type pattern in *Su(H)^*S*269*A*^* embryos, whereas embryos containing just the phospho-mimetic *Su(H)^*S*269*D*^* complement or lacking *Su(H)* like in the null mutant *Su(H)^*attP*^*, were characterized by a hyperplasia of the central and peripheral nervous system (Figures 2A–D).

Another Notch regulated process is the establishment of the dorso-ventral (d/v) boundary during wing imaginal development, which can be illustrated by the expression of the genes *cut* and *deadpan* (*dpn*). Both are expressed at high levels at the d/v boundary, whereas *dpn* is also detected at lower levels in intervein regions (Neumann and Cohen, 1996; Micchelli et al., 1997; Babaoglan et al., 2013; Chan et al., 2017). Both genes respond directly to Notch activation (Jack et al., 1991; Babaoglan et al., 2013; Chan et al., 2017). To address whether *cut* and *dpn* regulation is affected by the *Su(H)* phospho-site mutations, we performed a clonal analysis and compared the expression of Cut and Dpn protein in homozygous mutant versus wild type cell clones in wing disks. Again, *Su(H)^*S*269*A*^* mutant cells were like their wild type siblings, exhibiting normal expression levels of both, Cut and Dpn protein, i.e., indistinguishable from the *Su(H)^*gwt*^* wild type control (Figures 2E–H). In contrast, the *Su(H)^*S*269*D*^* mutation prevented Cut and Dpn expression at the d/v boundary, as did the null mutant *Su(H)^*attp*^*. In other regions of the wing disk, however, Dpn expression was expanded (Figures 2I–L). This suggests that mimicking phosphorylation of S269 in Su(H) interrupts the Notch-mediated transcriptional activation of target genes at the d/v boundary, but also Hairless-mediated silencing of *dpn* in other parts of the disk, failing its role as component of the Su(H)-H repressor complex.

In addition, we followed the activity of the Su(H)-dependent Notch responsive element *NRE:EGFP* along the d/v boundary (Saj et al., 2010). *NRE:EGFP* was not expressed in homozygous mutant wing disks of either *Su(H)^*attP*^* or *Su(H)^*S*269*D*^*, whereas no apparent difference to the control *Su(H)^*gwt*^* was observed in a *Su(H)^*S*269*A*^* homozygous background (Supplementary Figures 1A–D). Interestingly, a remnant expression of Wingless (Wg) was observed at the d/v boundary in *Su(H)^*S*269*D*^* mutant wing disks, suggesting that *Su(H)^*S*269*D*^* retains some Notch transducing activity depending on the context, and triggered target gene expression, respectively. In agreement with the *in situ* observation, no significant changes in the expression level of the two direct Notch target genes *E(spl)m3* and *E(spl)m8* (Krejčí and Bray, 2007; Housden et al., 2013) were detected between the control *Su(H)^*gwt*^* and *Su(H)^*S*269*A*^* by quantitative RT-PCR from wing imaginal disks, whereas *Su(H)^*S*269*D*^* mutants showed a reduced activity similar to a complete loss of *Su(H)* (Supplementary Figure 2). Moreover, Dpn expression was not lowered in *Su(H)^*S*269*D*^* and *Su(H)^*attP*^* wing disks (Supplementary Figure 2), which can be explained by the above finding of repression and de-repression of *dpn* in this tissue (Figures 2I–L).

Together these data indicate that the phospho-mimetic *Su(H)^*S*269*D*^* mutant generally affects Su(H) activity in Notch-dependent processes, i.e., activation as well as repression, most likely by interfering with the DNA-binding ability of the Su(H) protein. Hence, phosphorylation of Su(H) may serve to fine-tune Notch signaling activity in both directions in a context-specific manner. However, the phospho-deficient *Su(H)^*S*269*A*^* isoform did not enforce Notch signaling readout in any of the *in vivo* settings analyzed so far, despite the increased transcriptional activity of a Notch reporter in S2 cell culture (Nagel et al., 2017). This raised the question, if phosphorylation of Su(H) may occur in a more specific developmental setting, which we may uncover taking advantage of the phospho-deficient Su(H) isoform *Su(H)^*S*269*A*^*. In this case, we would expect an increase in Notch activity in a specific cell type or tissue, or during a distinct developmental process.

### Impaired Hematopoiesis in the *Su(H)^*S*269*A*^* Mutant Flies

**FIGURE 3:**
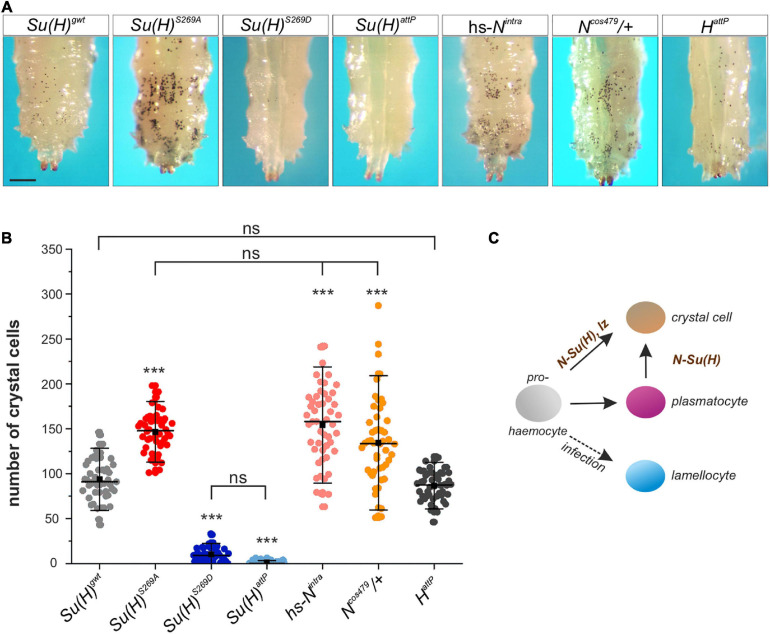
Larval crystal cell number is altered in *Su(H)* phospho-mutants. **(A)** Melanized larval crystal cells, visualized through the cuticle of the indicated genotypes. Note that all are homozygous but *N^*cos*479^*. Heat-blackened sessile cells from the last two segments are shown. Scale bar represents 250 μm for all panels. **(B)** Quantification of crystal cells from the last two segments in the indicated genotypes (*n* = 50 larvae for all genotypes). *Su(H)^*S*269*A*^*, hs-*N*^*intra*^ and *N^*cos*479^*^/+^ had a significantly increased number of crystal cells, whereas *H*^*attP*^ larvae displayed a level similar to the control. Crystal cells were barely detectable in *Su(H)^*S*269*D*^* and *Su(H)^*attP*^* homozygous mutant larvae. Every dot represents a measured larva. A black square represents the mean; horizontal bar represents the median, and error bars represent ± s.d. Statistical analysis by ANOVA two-tailed Tukey-Kramer approach: all samples but *H*^*attP*^ deviated with high significance from the control *Su(H)^*gwt*^* (***p < 0.001); in addition, not significant deviations are indicated (ns; *p* > 0.05). **(C)** Simplified scheme of hemocyte differentiation: the prohemocyte (gray) becomes either a crystal cell (beige), a plasmatocyte (purple) or, upon parasite infection, a lamellocyte (blue). Notch signaling controls crystal cell differentiation in the prohemocyte, and in the *trans*-differentiating plasmatocyte.

*Drosophila* Schneider S2 cells are derived from a primary culture of late stage embryos, likely from a hemocyte-like lineage (Schneider, 1972). Given that we have identified S269 phosphorylation on Su(H) in S2 cells, we speculated that this modification might be important in the context of blood cell development or differentiation. Three different types of mature hemocytes are present in *Drosophila* (reviewed in Letourneau et al., 2016). The majority are the phagocytic plasmatocytes. The second-most frequent cell type is the crystal cell, a blood cell type that produces prophenoloxidase (PPO) needed for an immune-related melanization process. Finally, the lamellocytes only appear upon bacterial or parasite infection (reviewed e.g. in: Evans et al., 2003; Vlisidou and Wood, 2015) (see scheme in Figure 3). A large proportion of larval plasmatocytes is sessile and resides within hematopoietic pockets attached to the integument, providing a microenvironment for survival, proliferation and differentiation (Makhijani et al., 2011; Leitão and Sucena, 2015). Crystal cells are post-mitotic, and arise by a Notch-dependent *trans*-differentiation process from plasmatocytes within these pockets (Rizki, 1957; Lanot et al., 2001; Leitão and Sucena, 2015). In a wild type larva, less than 5% of the hemocytes are crystal cells, but their number is considerably increased in a Notch gain of function background (Duvic et al., 2002). A quick and easy way to visualize mature larval crystal cells through the cuticle is by heating the larva, as this provokes the melanization cascade within the crystal cells (Rizki, 1957; Lanot et al., 2001). Consequently, the blackening of the crystal cells allows their counting directly through the cuticle. To address the consequences of the *Su(H)^*S*269^* phospho-site mutations on crystal cell development, we heated developmentally synchronized larvae and counted the crystal cell numbers in the last two posterior segments, where most of the crystal cells are sessile. As predicted for a loss of Notch activity, *Su(H)^*S*269*D*^* mutant larvae had very low numbers of crystal cells, similar to the null allele *Su(H)^*attP*^* (Figure 3). In the phospho-deficient *Su(H)^*S*269*A*^* homozygotes, the number of crystal cells was significantly increased, resembling *N^*cos*479^*^/+^ mutant larvae, which carry an additional copy of the *Notch* gene (Ramos et al., 1989) or larvae overexpressing the activated Notch-receptor (hs-*N*^*intra*^) shortly pulsed in the second larval stage (Figure 3). As H was shown to antagonize Notch activity in various developmental contexts (Maier, 2006), we additionally counted the number of crystal cells in *H* deficient larvae. Unexpectedly, the number of crystal cells was similar or rather lowered in comparison to the control (Figure 3 and Supplementary Figure 3), suggesting that H cannot repress Notch-mediated larval crystal cell differentiation. If this were the case, ubiquitous overexpression of H should strongly downregulate larval crystal cell numbers, which was not observed (Supplementary Figure 3). As *Su(H)^*S*269*A*^* mutant larvae constitute a gain of Notch activity in this developmental context, phosphorylation might be a different mechanism to regulate Su(H) activity during blood cell development, and hence to balance Notch signaling activity during hematopoiesis directly at the level of the transcription factor and hence, target gene transcription.

### Embryonic Crystal Cell Number Is Affected in *Su(H)* Phospho-Site Specific Mutants

**FIGURE 4:**
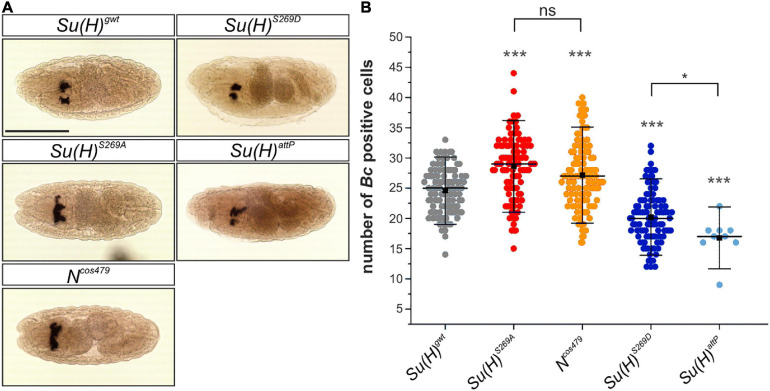
Embryonic crystal cell number is affected in *Su(H)* phospho-mutants. **(A)** Dorsal view of stage 14–16 embryos stained with a DIG-labeled *Bc* cDNA probe. The embryos were derived from germ line clones. Crystal cell precursors develop in bilateral clusters. Scale bar represents 200 μm for all panels. **(B)** Quantification of *Bc* positive cells in a total of 100 embryos of each genotype except for *Su(H)^*attP*^* (*n* = 9). Individual data points are indicated. Mean is shown as a black square, the median as horizontal bar. Error bars represent ± s.d. Highly significant differences are relative to control *Su(H)^*gwt*^* (in gray); ***p < 0.001; *p < 0.05; ns (not significant) *p* > 0.05; two-tailed Tukey-Kramer test. *Su(H)^*S*269*A*^* und *N^*cos*479/+^* mutant embryos harbored more *Bc* positive cells in the clusters, whereas *Su(H)^*S*269*D*^* and *Su(H)^*attP*^* embryos were characterized by lower numbers.

During a first wave of hematopoiesis embryonic crystal cells are formed. Initially, the crystal cell precursors remain clustered close to the site of their differentiation in the head mesoderm, before they eventually migrate to their final destination in the larva (Lebestky et al., 2000; Holz et al., 2003). To clarify whether crystal cell formation is affected in the *Su(H)* phospho-site specific mutant embryos, *in situ* hybridization was performed using a DIG-labeled probe against the crystal cell-specific gene *Black cells* (*Bc*), which encodes prophenoloxidase A1 (PPO-A1) (Milchanowski et al., 2004). We determined the number of *Bc* positive cells in late embryos within the two dorsal clusters in the head region. Earlier studies reported a lower number of crystal cells in *N* or *Su(H)* mutant embryos. Apparently, Notch signaling activity is not obligatory for embryonic crystal cell formation, but rather facilitates it (Lebestky et al., 2003; Bataillé et al., 2005). In agreement with the idea that phosphorylation impedes Su(H) activity, the number of crystal cells was reduced in embryos mutant for *Su(H)^*S*269*D*^* (20.2 ± 4.2) or *Su(H)^*attP*^* (16.8 ± 3.2) in comparison to the *Su(H)^*gwt*^* control (24.6 ± 3.7) (Figures 4A,B). Conversely, a significantly higher number of *Bc* positive crystal cells was obtained in either the phospho-deficient *Su(H)^*S*269*A*^* (28.6 ± 5.0) or *N^*cos*479^* embryos (27.3 ± 5.3) (Figures 4A,B). Thus, the phospho-deficient mutation of S269 in Su(H) had the same effect as increasing Notch activity already at early stages of crystal cell development, conforming to the idea of a novel phospho-specific regulatory mechanism of Notch-mediated hematopoiesis.

Together these results demonstrate a gain of Notch activity during embryonic and larval hematopoiesis in *Su(H)^*S*269*A*^* animals, presumably because Su(H) activity is no longer downregulated by phosphorylation through an upstream acting kinase. To test this hypothesis more directly, we performed immunoprecipitations with phospho-specific antibodies directed against parts of the Su(H) phospho-motif (phospho-S^∗^Q, see Figure 1A). Since the S269 phosphorylation is present in S2 cells, which are of embryonic origin (Schneider, 1972; Nagel et al., 2017), we used protein extracts from *Su(H)^*gwt*^* and *Su(H)^*S*269*A*^* embryos, respectively. Indeed, whereas all characteristic Su(H) protein bands were seen in precipitates of wild type embryos, only the larger protein species was robustly detected in the *Su(H)^*S*269*A*^* mutant embryos (Supplementary Figure 4). Altogether these data show that Su(H) protein is phosphorylated during embryogenesis presumably at several sites, most likely including S269.

### Phospho-Site Mutant Su(H), but Not H, Affects N-Dependent Crystal Cell Differentiation in the Larval Lymph Gland

**FIGURE 5:**
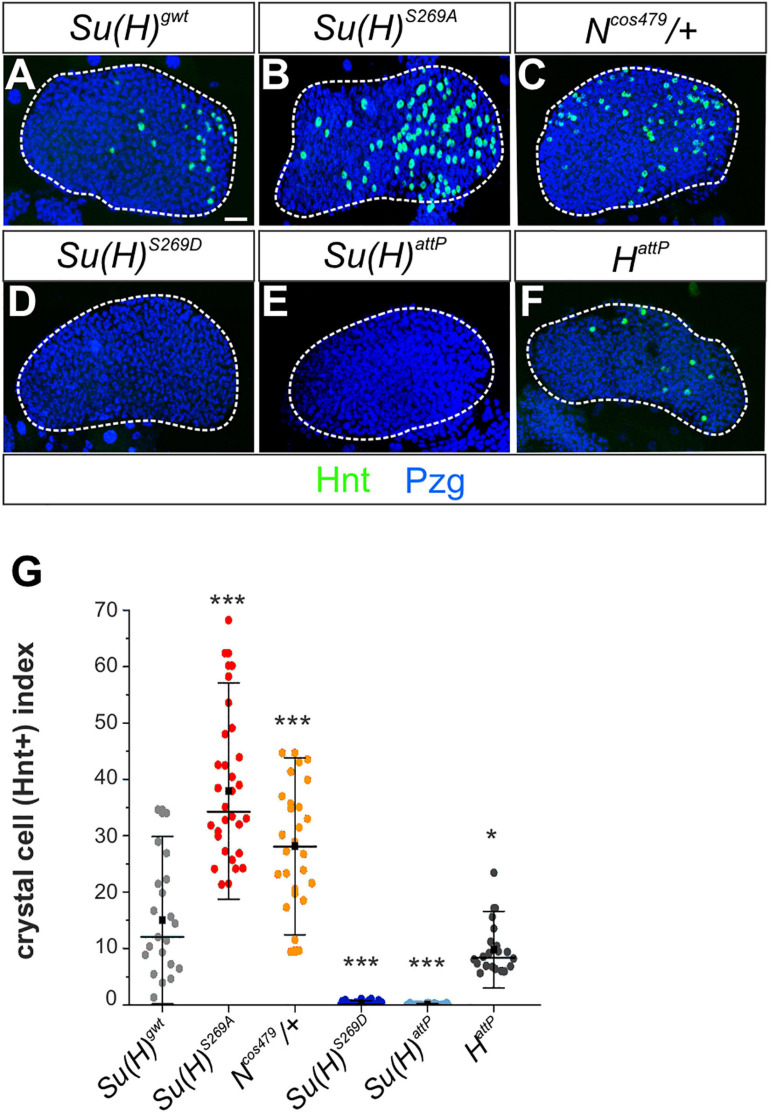
N-dependent lymph gland development is responsive to *Su(H)* phospho-site mutations. **(A–F)** Shown are primary lobes of third instar larval lymph glands stained with anti-Hnt (green) and anti-Pzg (blue). Crystal cell numbers were altered in *Su(H)* phospho-site mutant lymph glands relative to the control **(A,C,D)**. *N^*cos*479/^*^+^ **(B)** and *Su(H)^*attP*^* **(E)** were used for comparison to reflect a Notch gain- and loss background, respectively. **(F)** Lobes from *H*^*attP*^ mutants did not show an increased but rather a reduced number in Hnt positive cells. Scale bar represents 20 μm for all panels. **(G)** Ratio of Hnt^+^ cells per lobe relative to the lobe’s size. At least 20 lymph glands per genotype were scored. Each point represents one lobe. Mean is shown as a black square, the median is represented as horizontal bar. Error bars represent ± s.d. ***p < 0.001; *p < 0.05; ns (not significant) *p* > 0.05, two-tailed Dunnett’s test relative to control. Genotypes analyzed: *Su(H)^*gwt*^* (*n* = 21), *Su(H)^*S*269*A*^* (*n* = 30), *Su(H)^*S*269*D*^* (*n* = 28), *Su(H)^*attP*^* (*n* = 28), *N^*cos*479/^*^+^ (*n* = 26), *H*^*attP*^ (*n* = 20).

The larval lymph gland represents a second hematopoietic organ in the larva (reviewed e.g. in: Lebestky et al., 2000; Jung et al., 2005; Crozatier and Meister, 2007; Letourneau et al., 2016), where Notch signaling activity is required for crystal cell differentiation as well (Duvic et al., 2002; Lebestky et al., 2003; Terriente-Felix et al., 2013). In accordance, raising Notch signaling activity by overexpression of the Notch receptor itself, its ligand Serrate or of Su(H), induced a significant increase of crystal cell numbers in the lymph gland (Duvic et al., 2002; Lebestky et al., 2003; Blanco-Obregon et al., 2020). We hence wished to determine whether this other process of crystal cell formation might be influenced by phospho-site mutations in Su(H) as well. To this end, we analyzed the expression of Hindsight (Hnt) [also named Pebbled (Peb)] in developmentally synchronized third larval lymph glands, since *hnt* is a direct transcriptional Notch target gene in the context of crystal cell maturation (Benmimoun et al., 2012; Terriente-Felix et al., 2013; Tattikota et al., 2020). Quantitative measurements performed in at least 20 lymph glands each, demonstrated that *Su(H)^*S*269*A*^* (38 ± 12) and *N^*cos*479/^*^+^ (28 ± 10) mutant lobes exhibited a substantial increase in Hnt positive crystal cells compared to the *Su(H)^*gwt*^* (15 ± 9.7) control, whereas *Su(H)^*S*269*D*^* and *Su(H)^*attP*^* mutant glands were virtually devoid of mature crystal cells (Figures 5A–E). Moreover, the expression of the mature crystal cell marker PPO and the Notch-reporter *NRE:EGFP* were both increased in *Su(H)^*S*269*A*^* mutant lymph glands and decreased in the *Su(H)^*S*269*D*^* mutant, corroborating the above results (Supplementary Figure 5). We also noted a conspicuous increase in crystal cell size suggesting an effect on cell maturation as well (Terriente-Felix et al., 2013; Miller et al., 2017). Finally, although technically challenging, quantitative RT-PCR analyses were performed on whole isolated lymph glands for the expression of *hnt* and *PPO1*. Despite considerable variations observed in this tissue, a robust increase of *hnt* transcription was found in the *Su(H)^*S*269*A*^* lymph glands, and a very strong one of *PPO1* (Supplementary Figure 6), supporting the notion of an upregulation of Notch activity during crystal cell development in the phospho-deficient *Su(H)^*S*269*A*^* mutant. Collectively, these data suggest that phosphorylation of Su(H) might be a regulatory mechanism to specifically curb Notch activity in various aspects of hematopoiesis. The primary antagonist of Notch signaling activity in most developmental processes, however, is the corepressor Hairless, whose contribution to larval crystal cell development has remained obscure so far. To investigate the involvement of Hairless in this process, we assayed various crystal cell markers in *H*^*attP*^ mutant lymph glands. As already observed for sessile larval crystal cells, *H*^*attP*^ mutant glands did not show an increase in mature crystal cells but rather a slightly reduced number compared to the control (Figures 5F,G and Supplementary Figure 5). Thus, although being the best-characterized repressor of Notch signaling activity in *Drosophila*, we have no evidence that Hairless acts as Notch repressor during hematopoiesis in the context of larval crystal cell development.

### Regulation of CSL Activity by Phosphorylation Is Conserved Also in Mammals

**FIGURE 6:**
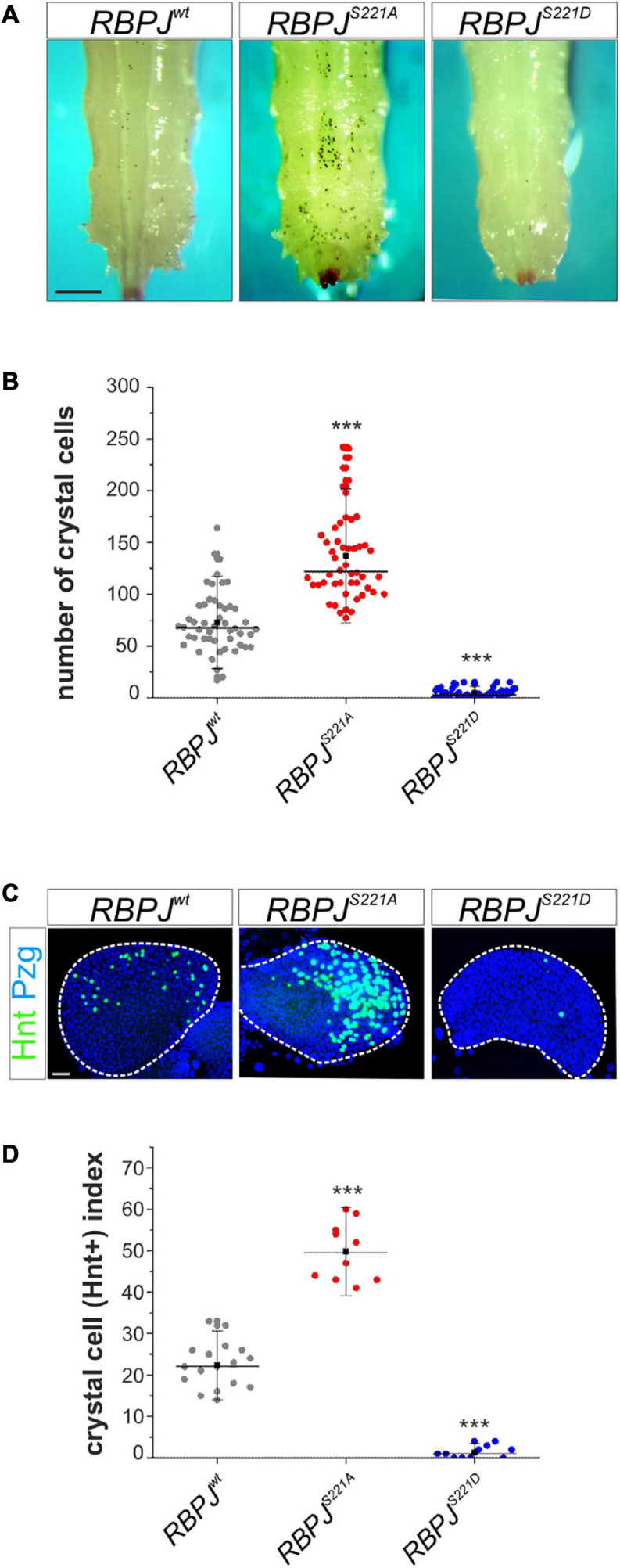
*Drosophila* crystal cell development is impeded in a murinized fly model. **(A)** Crystal cells, visualized by heating *RBPJ*^*wt*^, *RBPJ^*S*221*A*^*, and *RBPJ^*S*221*D*^* homozygous larvae. Scale bar represents 250 μm for all panels. **(B)** Quantification of melanized crystal cells within the last two segments of the given genotype (*n* = 50). *RBPJ^*S*221*A*^* had more, whereas *RBPJ^*S*221*D*^* larvae had reduced numbers of sessile crystal cells. Individual data points are indicated; mean is shown as a black square, the median as horizontal bar. Error bars represent ± s.d. ***p < 0.001; ns (not significant) *p* > 0.05, two-tailed Dunnett’s test relative to control. **(C)** Lymph glands from synchronized third instar larvae were stained with anti-Hnt (green) and anti-Pzg (blue) to detect mature crystal cells in the primary lobes (encircled by white dashed line). Scale bar represents 20 μm for all panels. **(D)** Crystal cell indices (number of Hnt positive cells relative to primary lobe size); individual data points are indicated. Mean is shown as a black square, the median as horizontal bar. Error bars represent ± s.d. ***p < 0.001. *RBPJ*^*wt*^ (*n* = 17), *RBPJ^*S*221*A*^* (*n* = 9), *RBPJ^*S*221*D*^* (*n* = 12).

Importantly, the site of Su(H) phosphorylation is entirely conserved in mammalian CSL orthologs. Moreover, this site was shown before to be phosphorylated *in vivo* (Rigboldt et al., 2011). Thus, we wanted to investigate whether the phospho-dependent regulatory mechanism is conserved also in mammals. First, we used a ‘murinized’ fly model, which we have recently established. In this fly strain, the mouse ortholog *RBPJ* was used to replace the endogenous *Su(H)* locus by genome engineering and shown to largely restore *Su(H)* essential functions (Gahr et al., 2019). To test our hypothesis that murine RBPJ, like Su(H), is subjected to a phospho-dependent regulation, we generated in addition the respective phospho-deficient and phospho-mimetic mutants, and established *RBPJ^*S*221*A*^* and *RBPJ^*S*221*D*^* fly strains by genome engineering. Subsequently, we analyzed the effects on Notch-dependent hematopoiesis. Confirming our assumption, heated *RBPJ^*S*221*A*^* larvae displayed an increased number of melanized crystal cells similar to the *Su(H)^*S*269*A*^* mutants. Accordingly, crystal cell numbers were strongly decreased in the phospho-deficient *RBPJ^*S*221*D*^* mutants, matching the *Su(H)^*S*269*D*^* homolog (Figures 6A,B). Moreover, Notch-dependent crystal cell development in the lymph gland was also affected: again, comparable to what has been observed in the respective *Su(H)* phospho-variants, phospho-deficient *RBPJ^*S*221*A*^* lobes possessed a larger number of Hnt-positive crystal cells than *RBPJ*^*wt*^ glands, whereas the phospho-mimetic *RBPJ^*S*221*D*^* variant was more or less devoid of Hnt positive crystal cells (Figures 6C,D). These results allow two conclusions. Firstly, they demonstrate that RBPJ transcriptional activity is likewise impaired by a phospho-mimetic mutation, presumably affecting DNA-binding similarly as in Su(H). Secondly, in the phospho-deficient *RBPJ^*S*221*A*^* mutant, Notch activity is increased in the context of Notch-dependent larval crystal cell development. We propose that RBPJ is recognized by the fly’s phosphorylation cascade as a cognate target, impeding Notch signal transmission accordingly. Hence, in the fly model, murine RBPJ is subjected to the same regulation as Su(H), demonstrating that CSL may be likewise targeted in mammalian cells, and that phosphorylation at S221 may be a way to downregulate Notch activity.

**FIGURE 7:**
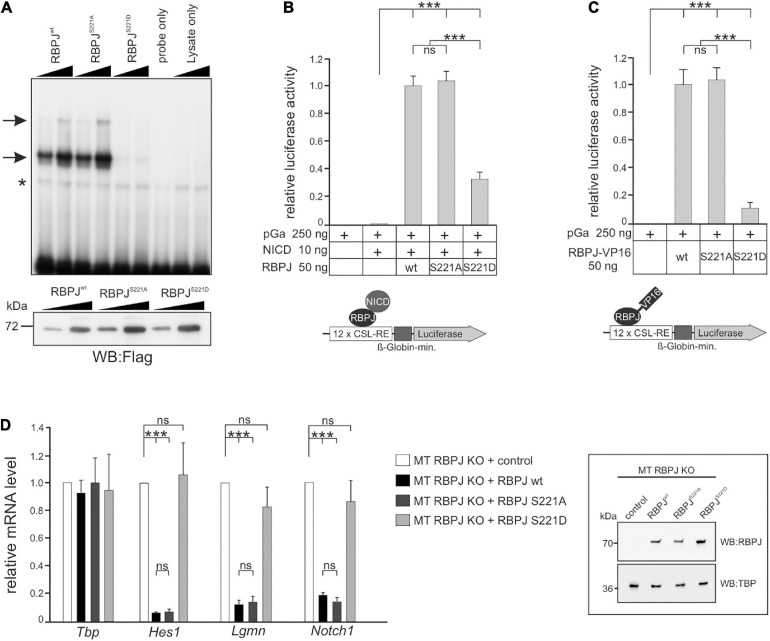
*RBPJ* phospho-mutants impact Notch activity in vertebrate cells. **(A)** Electromobility shift assays (EMSA) using *in vitro* translated Flag-tagged *RBPJ*^*wt*^ and *RBPJ* phospho-mutant proteins. Upper panel: DNA-binding complexes to an oligomeric duplex DNA probe with RBPJ binding sites are marked with arrows. The asterisk indicates unspecific background binding. Increasing amounts of TNT reticulocyte lysates (1 μl and 2 μl) were used as indicated. The double stranded oligonucleotides only and increasing amounts of reticulocyte lysates without *in vitro* transcription/translation reaction (lysates only) were used as controls. Lower panel: Relative abundance of *in vitro* translated Flag-tagged RBPJ fusion protein was tested by Western blotting using anti-Flag antibodies for detection. **(B)** Phospho-mimetic RBPJ^*S*221*D*^ displays impaired transcriptional activity in Notch-dependent luciferase-reporter assays in HeLa cells lacking endogenous *RBPJ*. Cells were co-transfected with the indicated plasmids of RBPJ variant and human Notch 1 intracellular domain (NICD) together with the CSL-RE luciferase reporter (pGA981/6). **(C)** Activation of the luciferase reporter by the given RBPJ variant fused to the VP16 transactivation domain. Note that lack of RBPJ^*S*221*D*^ activation is due to an impaired ability to bind to DNA. Luciferase activity is shown relative to activity of the reporter construct alone. Bars represent mean values from at least six independent experiments; error bars indicate standard deviation. **(D)** RBPJ phospho-mutant influences Notch target gene expression. Left panel: mouse hybridoma mature T-cells (MT) mutant for endogenous *RBPJ*, were used to re-introduce S221 *RBPJ* phospho-mutant variants as indicated, or empty vector (control). Total RNA was analyzed by qRT-PCR for the expression of the Notch target genes *Hes1*, *Lgmn*, and *Notch1*. Data shown represent the mean and standard of the mean of three independent experiments. Right panel: Western blots on MT cell nuclear extracts probed with an anti-RBPJ antibody. TATA-Box Binding Protein (TBP) was used as loading control. ANOVA two-tailed Tukey-Kramer test for multiple comparisons was applied for statistical analyses in panels **(B–D)** (***p < 0.001; [ns] not significant).

In order to test this hypothesis more directly, we addressed the activity of CSL phospho-site mutants in mammalian cells. As predicted, the introduction of a negative charge at position S221 diminished DNA-binding of murine RBPJ (Figure 7A). Next, we assayed the capacity of the RBPJ mutants in activating a Notch-reporter gene in the presence of NICD in HeLa^*RBPJ–KO*^ cells (Wolf et al., 2019). As shown in Figure 7B, RBPJ^*S*221*D*^ was strongly impaired in reporter gene activation, demonstrating the negative impact of the pseudo-phosphorylation, whereas RBPJ^*S*221*A*^ was similar to wild type RBPJ. Then RBPJ was coupled to the VP16 transactivation domain, which is expected to transactivate the reporter independent of NICD. Indeed, the RBPJ^*S*221*A*^-VP16 construct was indistinguishable in activating the reporter (Figure 7C), supporting the notion that phosphorylation on Ser 221 primarily acts on the DNA-binding of RBPJ. Moreover, we tested the activity of the respective RBPJ mutants in a mature T-cell line, in which endogenous RBPJ was depleted by CRISPR/Cas9 (Yuan et al., 2019). Reintroduction of wild type RBPJ leads to a full repression of Notch target genes *Hes1, Lgmn*, and *Notch1*, and likewise did RBPJ^*S*221*A*^, whereas RBPJ^*S*221*D*^ had lost activity as expected (Figure 7D). Together, these results indicate that a phospho-mimetic mutation at position S221 interferes with RBPJ’s activity. We suggest that also in mammalian cells dynamic S221-phosphorylation could be used as a switch to downregulate Notch signaling activity in a context specific manner.

## Discussion

### A Hairless-Independent Repression Mechanism of Notch Signaling Activity During *Drosophila* Hematopoiesis

As Notch signaling activity participates in a multitude of cell fate decisions, perturbation of the signal transmission as well as signal strength is linked to a multitude of human diseases, including the formation of solid tumors and leukemia (reviewed in: Louvi and Artavanis-Tsakonas, 2012; Bray, 2016). Thus, it is of utmost importance to understand the mechanisms underlying the regulation of Notch signaling activity in a context-specific manner. The model organism *Drosophila* has allowed to pioneer the Notch ‘interactome,’ i.e., the stunning plethora of genetic and molecular interactors identified in large-scale genetic and proteomic studies, revealing a complex and highly regulated network of genes modulating and fine-tuning Notch signaling activity (reviewed e.g. in: Hurlbut et al., 2007; Guruharsha et al., 2012; Ho et al., 2018). Downregulation of Notch activity is effectively mediated by repressors, which directly bind to the signal transducer CSL, thereby precluding target gene activation by Notch (reviewed e.g. in: Borggrefe and Oswald, 2009; Oswald and Kovall, 2018). In *Drosophila*, Hairless (H) is the most prominent and globally acting Notch antagonist, assembling a repressor complex together with Su(H) to silence Notch target genes in different developmental settings during imaginal development (Maier, 2006; Yuan et al., 2016). Our results, however, show that neither a gain nor a loss of *Hairless* function effected a repression or a de-repression of Notch-dependent crystal cell development during larval hematopoiesis, indicating that Hairless is not required in this process. In agreement, no significant change in gene expression of several well-known genes regulated by Notch during hematopoiesis, including the ones we have tested, was observed in *Hairless* depleted Kc cells (Chan et al., 2017). As this cell line, like Schneider S2 cells, is of hemocyte origin derived from embryos, and characterized by a hemocyte-like gene expression profile (Ramadan et al., 2007; Cherbas et al., 2011), a Hairless-independent mechanism of Notch repression must occur in this context. Instead, we propose phosphorylation of CSL as a means of confining Notch activity as illustrated by crystal cell formation during hematopoiesis.

### Dynamic Phosphorylation of Su(H) Increases the Plasticity of Notch Signaling Transduction

Besides the well-established model of Notch signal regulation based on the assembly of either a ternary activator complex or a repressor complex on target genes, an extensive network of crosstalk with other signaling cascades ensures a spatio-temporal and cell/tissue-type specific fine-tuning of Notch signaling intensity. A considerable fraction of the Notch ‘interactome’ includes factors that mediate posttranslational modifications such as phosphorylation, thereby targeting several Notch signaling members that positively or negatively change the biological outcome of Notch activity (Hasson et al., 2005; Lee et al., 2015; Carrieri and Dale, 2017; Ho et al., 2018). This includes also the central signal transducer Su(H) and RBPJ, respectively. Earlier studies have shown that MAPK-dependent phosphorylation of Su(H) attenuates Notch signaling activity in the fly, presumably by affecting the dynamics of the transition from activator to repressor complex formation (Auer et al., 2015). In contrast, p38 MAPK dependent phosphorylation of RBPJ in vertebrates accelerates its proteasomal degradation, thereby affecting its stability (Kim et al., 2011). Recently, we identified an additional phosphorylation occurring at S269 in the beta-trefoil domain (BTD) of Su(H), not interfering with either activator or repressor complex formation, but confounding DNA-binding (Nagel et al., 2017). Typical for CSL, Su(H) acts as a molecular switch in Notch signaling. Therefore, this modification potentially affects both, transcriptional activation and repression of Notch target genes, which was indeed supported by overexpression studies especially during wing development of the fly (Nagel et al., 2017). To circumvent potential dominant-negative effects that may result from excessive mutant protein in gain-of-function situations (Nagel et al., 2020), we instead replaced the wild type *Su(H)* gene with the phospho-specific S269 mutant variants at its native locus. As expected for a *Su(H)* mutant perturbed in DNA-binding, the phospho-mimetic *Su(H)^*S*269*D*^* variant affected the transmission of Notch signals in various developmental settings tested, however, still retained some DNA-binding activity *in vivo*. Accordingly, a weak activation of a reporter construct was obtained with both, Su(H)^*S*269*D*^ in *Drosophila* S2 cells (Nagel et al., 2017) as well as with RBPJ^*S*221*D*^ in HeLa cells (this study).

We expect that the altered DNA-binding of Su(H) contributes to the dynamics of Notch target gene regulation. Recent work from the lab of S. Bray has changed our view of CSL/Su(H) action: Su(H) does not appear as a strictly DNA-bound molecule with a stable residence. Instead, Su(H) binding at Notch target genes is a very dynamic process, changing from a transient DNA-occupancy in a Notch switched-off state toward an extended dwell time and enriched binding upon Notch receptor activation, resulting in a burst of target gene transcription (Krejčí and Bray, 2007; Gomez-Lamarca et al., 2018). Phosphorylation of Su(H) during this process is expected to strongly affect dwell time, and hence transcriptional output. Moreover, it is well established that Notch target enhancers differ in their responsiveness, both temporally and quantitatively. Accordingly, some target genes are turned on immediately after Notch activation, whereas others are delayed, and some genes respond several times more strongly than others do (Krejčí and Bray, 2007; Housden et al., 2013). One may envisage that the impeded DNA-binding of phosphorylated Su(H) is insufficient to activate the low and late responsive genes, whereas it may still activate the highly responsive targets to some level. In this case, phosphorylation is a mechanism to distinguish gene responses and to enhance the respective differences.

In addition, Notch target enhancers are also distinct with respect to their genomic situation, differing in number and spatial arrangement of Su(H) binding sites. Notably the paired Su(H) binding sites present in many Su(H) target genes allow cooperative binding of activator complexes and hence a markedly enhanced transcriptional response (Arnett et al., 2010; Hass et al., 2015). In contrast, Su(H) binding sites in a conventional orientation direct a linear response to Notch activity. If we assume that phosphorylation occurs at some, but not all Su(H) molecules at a given time, the outcome may differ dramatically at the different promoter types. Again, a linear response is expected for the conventional arrangement, depending on the number of phosphorylated Su(H) molecules and the residual DNA affinity. In contrast, if phosphorylation occurs in a dimeric activator complex at just one Su(H) molecule, cooperativity is lost, and simultaneously the peak of transcriptional activation is knocked down. Hence, we expect a non-linear, much stronger effect in genes regulated by paired Su(H) binding sites. On the other hand, preventing a possible phosphorylation, as mimicked by Su(H)^*S*269*A*^, a more stable and prolonged binding of activator complexes at the DNA would be expected, again increasing the differences in transcriptional output of promoters with unpaired versus paired Su(H) binding sites. Thus, phosphorylation at S269 adds to the DNA-binding dynamics of Su(H). We not only expect changes in the dynamics of Notch target gene activation, but likewise of repression. For example, *Su(H)^*S*269*D*^* mutant cell clones provide evidence for an impaired repressive activity, visualized in a de-repression of the Hairless susceptible target gene *dpn* in the wing disk and in agreement with the idea of a co-occupancy of Su(H) and H at the *dpn* intron enhancer (Chan et al., 2017). We may hence expect a dual response to Su(H) phosphorylation: in cells of high Notch activity, Notch target genes will become unresponsive, whereas in cells with low or no Notch activity, Notch target genes may be deregulated.

Neither the nuclear localization nor the stability of the Su(H) protein was affected in the phospho-specific S269 mutants. Instead, Su(H) phosphorylation acts at the level of DNA-binding and is hence, a very rapid, rigorous and effective way to curb Notch signaling outcome simply by impeding the accessibility to Notch target genes. Moreover, cofactor recruitment might be affected as well, despite the observation of an unrestricted activator and repressor complex assembly (Nagel et al., 2017). A kinase targeting Su(H) could in fact disrupt an ongoing Notch signaling process rapidly, even before the target gene promoter is reached, as well as thereafter. In other words, a specific signaling cascade could very effectively restrain NICD activity via the phosphorylation of Su(H).

Although novel for CSL transcription factors, phosphorylation as a means of regulating DNA-binding was previously described for other important transcription factors. This includes for example, the nuclear c-Myb oncoprotein (Lüscher et al., 1990), NF-kappaB (Vonderach et al., 2018), members of the Forkhead (Fox) family (Brent et al., 2008; Nie et al., 2013; Blane et al., 2018), as well as members of the Stat-family of transcription factors (Maiti et al., 2005). Interestingly, like CSL-mediated Notch signaling, most of these transcription factors regulate a great variety of biological processes. This specific posttranslational modification is, however, not part of their ‘canonical signal mechanism,’ but is instead a reaction toward misregulation, toward stress or is part of a spatio-temporal regulatory circuitry. We envisage that such mechanisms are also applicable for the transcription factor Su(H).

### Phosphorylation of Su(H) as a Means of Maintaining Blood Cell Homeostasis

Notch signaling activity regulates blood cell development in many respects, including the specification and maintenance of crystal cell precursors, their differentiation, maturation and survival. Hence, a tight regulation of Notch activity during hematopoiesis is of utmost importance for the maintenance of blood cell homeostasis. Interestingly, relative numbers of blood cell types vary little during development (Rizki, 1957), i.e., they must be strictly controlled. Both crystal cells and lamellocytes are generated through a process of transdifferentiation from plasmatocytes in the hematopoietic pockets (Honti et al., 2010; Stofanko et al., 2010; Leitão and Sucena, 2015). Moreover, both cell types originate from the lymph gland, albeit lamellocytes are induced only in response to parasite infection (reviewed in Small et al., 2014; Letourneau et al., 2016; Banerjee et al., 2019). Notch activity promotes crystal cell development, whereas it limits lamellocyte formation. In fact, lamellocytes form at the expense of crystal cells in response to parasitic wasp infection (Small et al., 2014). The mechanisms of Notch regulation in the context of blood cell homeostasis have remained largely elusive, notably regarding the restriction of Notch activity. Crystal cell precursor formation depends on Notch receptor activation by its ligand Serrate (Lebestky et al., 2003; Leitão and Sucena, 2015), whereas maturation and survival of crystal cells relies on ligand-independent mechanisms of Notch activation, involving the regulation of Notch receptor expression and stability (Mukherjee et al., 2011; Terriente-Felix et al., 2013; Miller et al., 2017). We propose Ser269-phosphorylation of Su(H) in the context of hematopoiesis as a means to maintain blood cell homeostasis. This process appears independent of Hairless that otherwise functions as the major antagonist of Notch in most developmental processes. Instead, phosphorylation of Su(H) may allow to block Notch activity downstream of receptor activation, i.e., ligand-dependent Notch activity as well as ligand-independent Notch activity, be it at the level of Notch expression and stability or at the level of Notch target gene activation in conjunction with other factors. Decreasing Notch activity in Notch-responsive cells is expected to reduce crystal cell numbers by interfering with their specification in the lymph gland as well as their determination from plasmatocytes, in addition to their maturation and survival. Consequently, a shift to plasmatocytes at the expense of crystal cells might occur, allowing to regulate blood cell type proportions. Maintenance of homeostasis may involve Su(H) phosphorylation in a specific subset of hemocytes to allow immediate activation of Notch signaling by a phosphatase, inducing crystal cell fate if needed. Moreover, a downregulation of Notch activity might help to combat to parasitic wasp infestation by encouraging the formation of combatting lamellocytes (Small et al., 2014), and could be triggered very quickly and efficiently via the phosphorylation of Su(H). Blood cell homeostasis is influenced by many signals including developmental timing, nutrition, olfaction and hypoxia (reviewed in Banerjee et al., 2019). Accordingly, cross-talk between Notch and various other signaling pathways is expected. Phosphorylation of Su(H) may be one aspect of such a cross-talk, as it presents a way of blocking Notch activity in an immediate and reversible manner. As the phospho-deficient *Su(H)^*S*269*A*^* mutant is homozygous viable and inconspicuous with regard to developmental timing, Notch dependent processes during embryonic neurogenesis as well as wing development, we can rule out the idea of having a general ‘overactivated’ Notch signaling mutant in hand. Phosphorylation of Su(H) is expected to influence blood cell homeostasis by confining Notch signaling output, presenting a prime target for a respective kinase during the cross-talk with relevant signaling pathways.

Our ‘murinized’ fly model that carries the respective RBPJ phospho-mutants in place of Su(H), displays likewise phenotypes, suggesting phosphorylation as a more general mechanism to regulate DNA-binding affinity of CSL. Interestingly, the human CSL ortholog is specifically phosphorylated at the corresponding position Ser195 during embryonic stem cell differentiation (Rigboldt et al., 2011), in line with our idea of maintaining blood cell homeostasis by regulating Notch activity through CSL phosphorylation. Accordingly, replacing *RBPJ*^*wt*^ with the *RBPJ^*S*221*D*^* phospho-mimetic variant clearly disturbed RBPJ’s ability to act as repressor in a mature T-cell line. Moreover, *RBPJ^*S*221*D*^* induced a strongly decreased transcriptional response together with NICD in a luciferase based test system. Therefore, in vertebrates like in *Drosophila*, the phospho-mimetic variant of CSL derogated the transcriptional outcome of Notch signaling, presumably by interfering with DNA-binding on target genes, whereas the phospho-deficient RBPJ^*S*221*A*^ variant retained a wild type level of activity. Again, as already observed in our *Drosophila* model, no general boost in Notch activity was observed in line with a context-specific mechanism. Altogether our data support a model, whereby phosphorylation of Su(H) is a novel, and perhaps conserved means of tissue-specific Notch silencing that appears independent of the corepressor *Hairless*. A major challenge in the future will be to determine the responsible kinase(s) involved and unravel the cross-talk that regulates Notch in the maintenance of blood cell homeostasis.

## Materials and Methods

### Generation of Su(H)^*S*269^ and RBPJ^*S*221^ Phospho-Mutant Constructs and Fly Lines

Genome engineering was used to replace the *Su(H)* genomic region with the phospho-specific *Su(H)^*S*269*A*^* and *Su(H)^*S*269*D*^* or murine *RBPJ^*S*221*A*^* and *RBPJ^*S*221*D*^* variants. To obtain the respective *Su(H)* DNA, a 734 bp *Nar*I fragment within the second exon of pBT-*Su(H)^*gwt*^* was exchanged by a *Nar*I fragment containing the phospho-mutant variant (described in Nagel et al., 2017), thereby yielding pBT-*Su(H)^*S*269*A*^* and pBT-*Su(H)^*S*269*D*^* genomic subclones. Finally, they were shuttled as *Bam*HI/*Xho*I fragments into *Bgl*II/*Xho*I opened pGEattB^*GMR*^ vector (Huang et al., 2009). To obtain the respective *RBPJ* DNA, substitution mutations (serine 221 by alanine or aspartic acid) were introduced into the pBT ΔNEP RBPJ subclone (Gahr et al., 2019), by PCR-mutagenesis using sequence specific mutagenesis primer pairs. The *Bam*HI/*Xho*I fragment harboring the first intron of *Su(H)* and the entire mutated mouse *RBPJ* cDNA was shuttled into *Bgl*II/*Xho*I opened pGE-attB^*GMR*^. All four constructs were each injected in embryos containing the founder line *Su(H)^*attP*^* (Praxenthaler et al., 2017) and the vasa-Phi C31 integrase (BL40161), to insert the phospho-mutant variants by site specific recombination. The *white*^+^ marker gene and vector sequences were finally deleted as described in Praxenthaler et al. (2017) and Gahr et al. (2019). The phospho-specific *Su(H)* and *RBPJ* mutants were established and balanced with CyO-GFP. The obtained mutants were firstly confirmed by PCR and diagnostic restriction digests and finally sequence verified (Macrogen Europe, Amsterdam, Netherlands).

### Generation of Germ Line- and Somatic Clones

To induce Flp/FRT-based mutant cell clones, *Su(H)^*S*269*A*^* and *Su(H)^*S*269*D*^* alleles were recombined with FRT40A and combined with a heat-inducible flippase on the X chromosome (*hs-flp^1^.^22^*; *Su(H)^∗^ FRT40A*/CyO). To obtain female germline mutants for the analysis of maternal effects, females were crossed with *OvoD1 FRT40A*/CyO males (BL2121). Larval offspring (48 h after egg laying [AEL]) were heat-shocked for 1 h at 37°C, followed by a second heat-shock (96 h AEL) for 30 min. Female offspring without CyO balancer were selected and crossed with wild type (OregonR1) males. As OvoD disrupts oogenesis, only females in which FRT-mediated recombination was induced are able to produce mature eggs. These eggs were collected in a daily manner and further processed for antibody staining and *in situ* hybridization.

To induce Flp/FRT based somatic mosaics, *hs-flp^1.22^*; *Ubi-GFP FRT 40A* flies were crossed with *Su(H)^∗^ FRT40A*/CyO-GFP flies. Early second instar larval offspring (app. 48 h AEL) were heat-shocked for 1 h at 37°C and further developed at 25°C. Third larval instars were prepared for antibody staining.

### Immunochemistry (Staining of Embryonic and Larval Tissues, Western Blot)

#### Anti-HRP Staining of Embryos

Dechorionated embryos were fixed in reaction tubes with 500 μl *n*-heptane and 500 μl formaldehyde (10% in PBS) for 15 min on a rotating wheel. After removing the lower phase an equal amount of cooled (−80°C) methanol was added and shaken rigorously for 30 s to remove the vitelline membrane. After removing the upper phase, the devitellinized embryos were washed three times with methanol. At this point the embryos could be stored in methanol at −20°C. Embryos were rehydrated by reducing methanol concentration in a step-wise manner (80/60/40/20%) with PBS. After washing three times with PBT (PBS, 0,1% Tween 20) the embryos were pre-incubated with 4% NGS in PBT for 1 h at RT. Embryos were stained with anti-HRP-FITC labeled antibody (Jackson Immuno Research Laboratories) at 8°C overnight. Embryos were washed four times with PBT 15 min each and mounted in Vectashield (Vector Laboratories).

#### Staining of Larval Disks

Imaginal disks from third instar larvae were dissected in PBS and fixed in 4% paraformaldehyde for 20 min while gently rocking. The disks were washed several times with PBX (PBS with 0.3% Triton X-100), pre-incubated with 4% NDS and stained with primary antibodies overnight at 8°C. After several washes with PBX, tissues were pre-incubated with 4% NDS before secondary antibodies, coupled with fluorescence dyes, were added. The incubation was either overnight at 8°C or 2–3 h at room temperature. After washing with PBX, separated wing disks were mounted on microscope slides in Vectashield (Vector Laboratories).

#### Staining of Larval Lymph Glands

To obtain a synchronized larval population, females were allowed to lay eggs in intervals of 4 h. Lymph glands from synchronized batches were dissected in ice-cold PBS and fixed in 4% paraformaldehyde for 25 min. Tissues were permeabilized by three washings with PBX and blocked with 4% NDS or NGS in 0.3% PBX for 45 min – 1 h. Incubation with primary antibodies was carried out at 8°C overnight. Lymph glands were washed four times 15 min each with PBX and blocked with 4% NDS or NGS for 45 min. Secondary antibodies were added at the appropriate dilution and incubated either overnight at 8°C or 2–3 h at RT. Tissue was embedded on microscope slides in Vectashield (Vector Laboratories).

#### Acquisition and Documentation of Stained Tissues

Fluorescently labeled tissues were documented with a Zeiss Axioskop coupled with a BioRad MRC1024 confocal microscope, using LaserSharp 2000 software. Images were acquired as confocal sections using the same settings within each set of experiments. Confocal data was gained at least from three independent experiments to ensure reproducibility. On average more than 10 embryos or imaginal tissues with cell clones from each genotype were analyzed. For quantification of cell types in lymph glands at least 20 were evaluated using the cell counter function of *Fiji* (*Image J*) software. Freehand selection allowed to encircle the tissue to determine the size (in pixel) by selecting the area measurement tool. Indices encounter the number of cells in relation to the size of tissue and were determined by: number of cells in tissue/area size (in pixel) ×10000.

#### Quantification and Immunoprecipitation of Su(H) Protein by Western Blotting

Ten homozygous third instar larvae of each genotype were homogenized and treated according to Praxenthaler et al. (2017) and Gahr et al. (2019). The blots were cut to detect Su(H) protein (Rabbit anti-Su(H), Santa Cruz Biotechnology) and betaTubulin (Mouse anti-beta Tubulin, Developmental Studies Hybridoma Bank) as loading reference from the same lane. Goat secondary antibodies coupled with alkaline phosphatase (Jackson Immuno Research Laboratories) were used for detection. To statistically quantify the signals, *n* = 3 blots from three independent experiments were analyzed with *Image J* gel analysis program. Su(H) protein levels from phospho-mutants were compared with those of Su(H)^*gwt*^. Significance was tested using ANOVA Tukey-Kramer approach for multiple comparisons.

Immunoprecipitations were performed essentially as outlined before (Harlow and Lane, 1999; Nagel et al., 2001). Briefly, about 50 mg of embryos from overnight collections were extracted in 200 μl of binding buffer (Goodfellow et al., 2007). Protease and phosphatase inhibitor cocktails (ROCHE complete ULTRA, and PhosSTOP; Roche) were added to all buffers according to the manufacturer. 45 μl of protein A Sepharose beads (Roche) were pre-incubated with 10 μl of phospho-ATM/ATR substrate (S^∗^Q) mAB (CST #9607) at 4°C overnight, mixed with protein extracts and immuno-complexes allowed to form for 2 h at 4°C. After several washes in binding buffer, the precipitates were collected in 50 μl SDS-loading buffer, and 18 μl used per lane for subsequent detection with Su(H) antibodies (Santa Cruz Biotechnology), compared to 0.7% of the input. Goat secondary antibodies coupled with alkaline phosphatase (Jackson Immuno Research Laboratories) were used for detection.

#### *In situ* Hybridization of Embryos and Quantification of Embryonic Crystal Cell Precursors

Embryonic hematopoiesis was analyzed at stage 14–16 embryos to compare the amount of crystal cell precursors in bilateral clusters in the head mesoderm. To this end, females were allowed to lay eggs for 3 h on apple juice plates at 25°C with a freshly paste of yeast. After 10 h at 25°C, embryos were treated and collected in methanol as described for antibody staining above. *In situ* hybridization was performed according to the protocol of Tautz and Pfeifle, 1989. As probe, Dig-labeled cDNA from PPO1 (GH04080) was used. PPO1 positive cells were counted on embedded embryos with a Zeiss Axioscope coupled to a Pixera Pro300D camera using iWorks 2.0 software. *Su(H)^*gwt*^*, *N^*cos*479^* and *Su(H)^*S*269*A*^* embryos were gained from homozygous females, whereas *Su(H)^*S*269*D*^* and *Su(H)^*attP*^* mutant embryos were derived from mothers with germ line clones. With exception of *Su(H)^*attP*^* (*n* = 9), *n* = 100 embryos were analyzed for each genotype.

### qRT-PCR Analysis With *Drosophila* Tissue

Quantitative RT-PCR was performed on four biological and two technical replicates of each genotype using either 40 isolated wing disks or lymph glands. Poly (A)^+^ RNA preparation, cDNA synthesis and real-time qPCR were performed as outlined earlier (Praxenthaler et al., 2017; Kober et al., 2019). As internal reference genes, *cyp33*, *rp49* and *Tbp* were used. Relative quantification of the data was done with micPCR software Version 2.10.0, based on REST (Pfaffl et al., 2002). Expression values *p* < 0.05 were considered to be statistically significant. All kits, oligonucleotides and software used for these approaches are summarized in Supplementary Table 1.

### Developmental Assays

To determine developmental timing as well as adult hatching rates, all *Su(H)* variants were balanced over the CyO-GFP balancer chromosome. To avoid overcrowded conditions 15 virgin females were crossed with 10 males each and crosses were subjected to fresh food vials every 8 h at 25°C. The number of homozygous pupae and hatching rates of *Su(H)^∗^* homozygotes were counted and normalized to the total of counted flies. Results from 10 independent crosses were enumerated.

### Melanization of Larval Crystal Cells

To obtain synchronized developed larvae, flies were allowed to lay eggs for 4 h. Late third instar larvae were shortly washed in PBS and batches of five larvae were placed in PCR tubes filled with water. Larvae were heated at 60°C for 15 min to induce the melanization of crystal cells. For quantification, the number of crystal cells in the last two segments of each larva was counted using *Image J*.

### Statistical Analysis

All collected data were statistically evaluated: calculation of the average mean and standard deviations (s.d.) were recorded via Microsoft Excel; statistical significance was evaluated by using a two-tailed analysis of variance (ANOVA) approach for multiple comparisons according to Dunnett’s Test and Tukey-Kramer’s Honestly Significance Difference. Statistical graphs were created with Origin^®^ 2018b software (OriginLab Corporation).

### *In vitro* Protein Translation (TNT-Assay)

*In vitro* protein translations were performed using the TNT-assay from Promega according to the manufacturer’s instructions. After *in vitro* translation of *RBPJ*^*wt*^ and phospho-mutant proteins, expression was monitored by Western blotting (primary antibody: anti-Flag, Merck; secondary antibody: HRP-conjugated sheep anti-mouse IgG, GE Healthcare; see Supplementary Table 1).

### Electro Mobility Shift Assay (EMSA)

Reticulocyte lysates (1 μl and 2 μl) from *in vitro* translations were used for electromobility shift assays (EMSAs). Binding reaction was performed in a buffer consisting of 10 mM Tris-HCl (pH 7.5), 100 mM NaCl, 0.1 mM EDTA, 0.5 mM DTT and 4% glycerol. For binding reaction, 2 μg poly(dI-dC) (GE Healthcare) and approximately 0.5 ng of ^32^P-labeled oligonucleotides were added. The sequence of the double-stranded oligonucleotide FO-233 (Supplementary Table 1) corresponds to the two RBPJ-binding sites within the EBV TP-1 promoter. DNA-protein complexes were separated using 5% polyacrylamide gels with 1x Tris-glycine-EDTA at room temperature. Gels were dried and exposed to X-ray films (GE Healthcare).

### HeLa Cell Culture Experiments and Luciferase Assay

Generation and characterization of the RBPJ depleted HeLa cells was shown previously (Wolf et al., 2019). HeLa^*RBPJ–KO*^ cells were cultivated in Dulbecco’s Modified Eagle Medium (DMEM, GIBCO) supplemented with 10% fetal calf serum (FCS) and penicillin/streptomycin. Cells were seeded in 48-well plates at a density of 20 × 10^4^ cells. Transfection of the reporter construct pGa (12 × CSL-RE-Luc) together with expression constructs was performed with Lipofectamine 2000 reagent (Thermo Fisher) using 250 ng of reporter plasmid alone or together with various amounts of expression plasmid (as given in the figure). After 24 h luciferase activity was determined from at least six independent experiments from 20 μl of cleared lysate. Measurements were performed using a LB 9501 luminometer (Berthold) and the luciferase assay system from Promega.

### Rescue-Experiments With RBPJ^*wt*^ or RBPJ-Phospho Mutants in Mouse Hybridoma Mature T-Cells Deficient for RBPJ

CRISPR/Cas9-mediated *RBPJ* depleted mouse hybridoma mature T-cells were generated as previously described (Yuan et al., 2019). T-cell lines stably expressing RBPJ^*wt*^, phospho-deficient (S-to-A) or phospho-mimetic (S-to-D) RBPJ-mutant depleted MT-cells were generated as follows: 5 × 10^6^ Phoenix^TM^ cells were seeded and 24 h later they were transfected with the retroviral plasmid DNA without insert (control) or containing *RBPJ*^*wt*^, *RBPJ^*S*221*A*^* or *RBPJ^*S*221*D*^*. Briefly, 20 μg of DNA were mixed with 860 μl of H_2_O and 120 μl of 2x HBS buffer (50 mM HEPES pH 7.05, 10 mM KCl, 12 mM Glucose, 280 mM NaCl, 1.5 mM NaHPO_4_) while vortexing and the solution was incubated 20 min at room temperature. In the meantime, 25 μM Chloroquine solution (Sigma-Aldrich) was added to the Phoenix^TM^ cells (1 μl/ml) and the cells were incubated for 10 min. The DNA solution was added to the cells and 12 h later the medium was replaced. After 24 h of incubation, the medium containing the retroviral suspension was filtered and Polybrene (Sigma-Aldrich) solution was added. Fresh medium was added to the Phoenix^TM^ cells that were maintained in culture for further infections. The retroviral solution was used for spin infection of MT cells by centrifuging 45 min at 1800 rpm at 37°C. In total, four spin infections were performed over 2 days. Positively infected cells were selected with Blasticidin (Gibco).

### Cell Culture

Mouse hybridoma mature T (MT)-cells were grown in Iscove’s Modified Dulbecco Medium (IMDM, Gibco) supplemented with 2% FCS, 0.3 mg/l peptone, 5 mg/l insulin, essential amino acids and penicillin/streptomycin. Cells were grown at 37°C with 5% CO_2_. Phoenix^TM^ packaging cells (Orbigen, Inc., San Diego, CA, United States) were cultivated in Dulbecco’s Modified Eagle Medium (DMEM, Gibco) supplemented with 10% FCS and penicillin/streptomycin.

### Preparation of Protein Extracts and Western Blotting From Mouse Hybridoma Mature T-Cells

The nuclear extract (NE) from MT cells overexpressing the RBPJ constructs or control cells containing empty vector was prepared as follows. Briefly, 10 × 10^6^ cells were washed with PBS and resuspended in 200 μl of Buffer 1 (10 mM HEPES pH 7.9, 10 mM KCl, 0.1 mM EDTA, 0.1 mM EGTA, 1 mM beta-mercaptoethanol, supplemented with PMSF). The cell suspension was incubated 10 min on ice, 5 μl of 10% NP-40 were added and mixed by vortexing. After 10 s of centrifugation at 13000 rpm at 4°C, the nuclei pellet was washed twice in 500 μl of Buffer 1 and resuspended in 100 μl of Buffer 2 (20 mM HEPES pH 7.9, 400 mM NaCl, 1 mM EDTA, 1 mM EGTA, 1 mM beta-mercaptoethanol, supplemented with PMSF). After 20 min of incubation on ice, the nuclei suspension was centrifuged 10 min at 13000 rpm at 4°C. and the supernatant was collected for further analysis. Protein concentration was measured by Bradford assay (BioRad) and samples were boiled after adding SDS-polacrylamide gel loading buffer. Samples were resolved by SDS-Page and analyzed by Western blotting using antibodies against RBPJ (CST #5442) or TBP (Abcam). Briefly, membrane for anti-TBP Western blotting was blocked in 5% milk, 1x TBS, 0,1% Tween-20 (TBS-T) and incubated over night at 4°C with primary antibody diluted in 5% milk, TBS-T. Next, membrane was washed in TBS-T (5 × 5 min), incubated for 1 h at room temperature with secondary antibody against mouse (Cell Signaling) diluted in 5% milk TBS-T and washed again in TBS-T (5 × 5 min). Membrane for anti-RBPJ Western blotting was blocked in 5% milk, 1x TBS and incubated over night at 4°C with primary antibody diluted in 5% BSA, 1x TBS, 0.3% NP40. Next, membrane was washed in 1x TBS, 0.5 M NaCl, 0.5% Triton X-100 (3 × 15 min), incubated for 1 h at room temperature with secondary antibody against rabbit (Jackson ImmunoResearch Laboratories) diluted in 5% BSA, 1x TBS, 0.3% NP-40 and washed in 1x TBS, 0.5 M NaCl, 0.5% Triton X-100 (3 × 15 min). All membranes were finally incubated with ECL solution and chemiluminescence was detected with CCD-Camera FUSION-FX7.

### Gene Expression Analysis as Measured by qRT-PCR in Mouse Hybridoma Mature T-Cells

Total RNA was purified using TRIzol reagent accordingly to manufacturer’s instructions. 1 μg of RNA was reverse transcribed in cDNA using random hexamers and M-MuLV reverse transcriptase (New England Biolabs). Quantitative PCRs were assembled with Absolute QPCR ROX Mix (Thermo Scientific, AB-1139), gene-specific oligonucleotides and double-dye probes and analyzed using StepOne Plus Real Time PCR system (Applied Biosystems). Data were normalized to the housekeeping gene Hypoxanthine phosphoribosyltransferase (Hrpt).

## Data Availability Statement

The original contributions presented in the study are included in the article/Supplementary Material, further inquiries can be directed to the corresponding author.

## Author Contributions

SD, PH, LF, BG, AN, FO, AT, HS, and MZ performed the experiments. TB, PH, LF, BG, AN, FO, and AT provided methodology. TB, LF, BG, AN, and FO provided resources. LF, BG, AN, FO, HS, AT, and MZ analyzed and visualized the data. TB, AN, and FO supervised the project and acquired funding. AN conceptualized the project and drafted the manuscript. All authors contributed to the article writing and approved the submitted version.

## Conflict of Interest

HS is currently employed at AstraZeneca. All research presented in this manuscript was completed before any commercial affiliations took place. The remaining authors declare that the research was conducted in the absence of any commercial or financial relationships that could be construed as a potential conflict of interest.
