# Supplementary material for: Phospho-Site Mutations in Transcription Factor Suppressor of Hairless Impact Notch Signaling Activity During Hematopoiesis in Drosophila
Source: Front Cell Dev Biol. 2021 Apr 14;9:658820. doi: 10.3389/fcell.2021.658820 (PMC8079769; doi:10.3389/fcell.2021.658820)
Supplement: Supplementary file 1 [file Data_Sheet_1.PDF]

Supplementary Figures 1-6

Supplementary Table 1

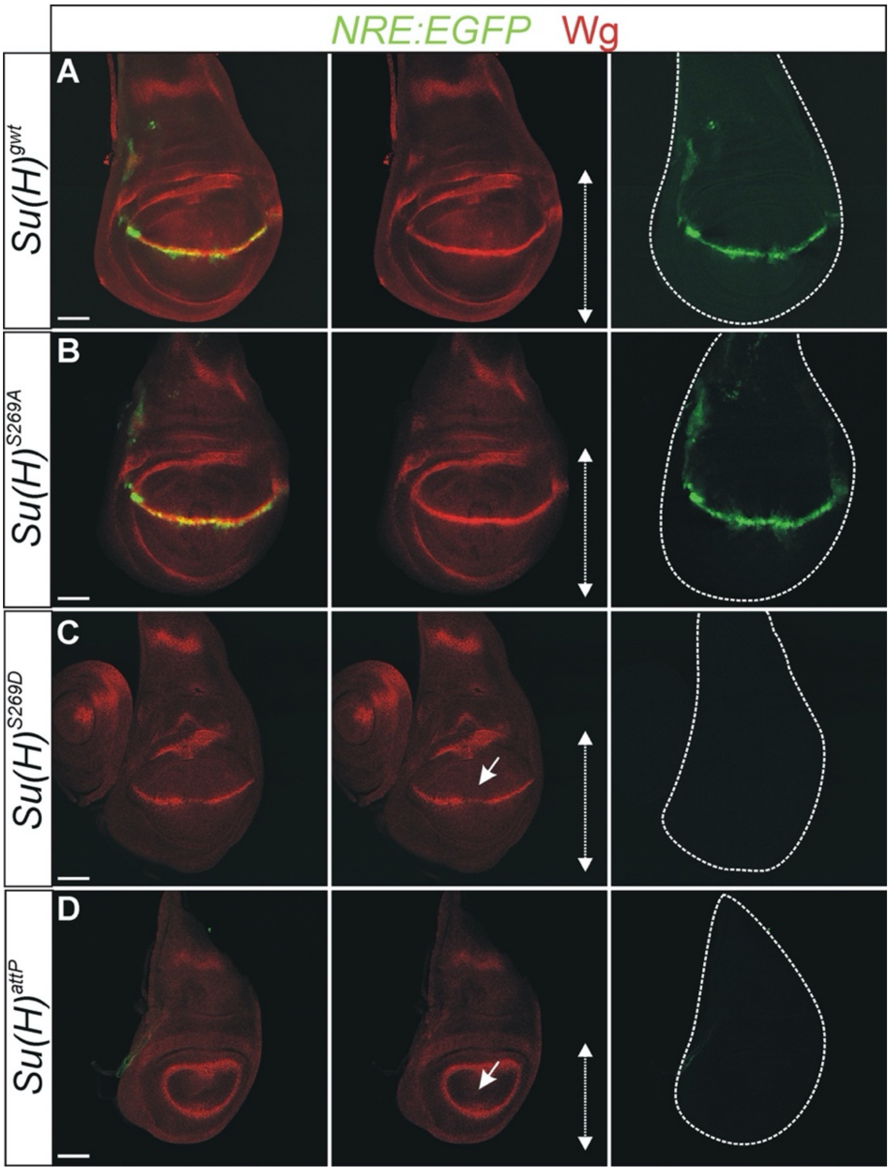

Supplementary Figure 1. *NRE:EGFP* and Wg expression in *Su(H)* phospho-mutants

(**A-D**) *NRE:EGFP* reporter (green) and Wg (red) expression is shown. Readout is similar comparing $Su(H)^{gwt}$ (**A**) and $Su(H)^{S269A}$ (**B**). By contrast, $Su(H)^{S269D}$ (**C**) and $Su(H)^{attP}$ (**D**) mutants are devoid of *NRE:EGFP* expression, whereas Wg expression at the d/v boundary is reduced in $Su(H)^{S269D}$ (**C**, arrow) and absent in $Su(H)^{attP}$ (**D**, arrow). Wing blade size is similar in $Su(H)^{gwt}$ and $Su(H)^{S269A}$ (**A,B**), slightly reduced in $Su(H)^{S269D}$ (**C**) and very small in $Su(H)^{attP}$ (**D**) (double headed arrows). Scale bar: 100 μm in all panels.

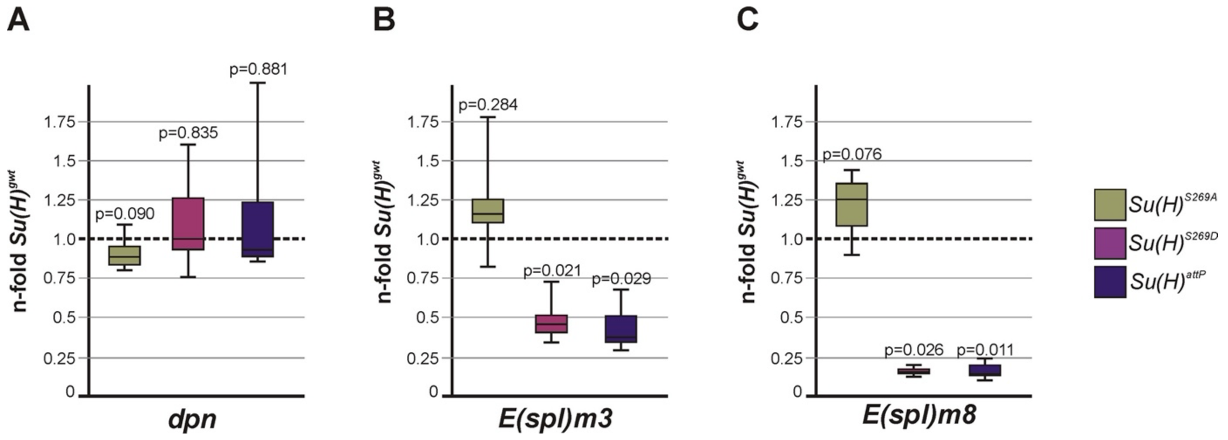

Supplementary Figure 2. qRT-PCR analyses of Notch target genes from isolated wing discs Transcript levels of the Notch target genes *dpn* (**A**), *E(spl)m3* (**B**) and *E(spl)m8* (**C**) were quantified by qRT-PCR from 40 isolated wing discs relative to the control $Su(H)^{gwt}$. *Tbp* and *cyp33* were taken as reference genes. Data were gained from four biological and two technical replicates. No statistically significant changes were observed between $Su(H)^{S269A}$ and $Su(H)^{gwt}$, whereas in $Su(H)^{S269D}$ and $Su(H)^{attP}$ mutant discs *E(spl)m3* and *m8* transcripts are reduced. *Dpn* transcripts are unaltered in the mutants, presumably due to the decrease of normal H-mediated repression. Mini-max depicts 95% confidence, median corresponds to expression ratio. The p-values are given above each bar. Significance was tested using PFRR from REST ($p<0.05$).

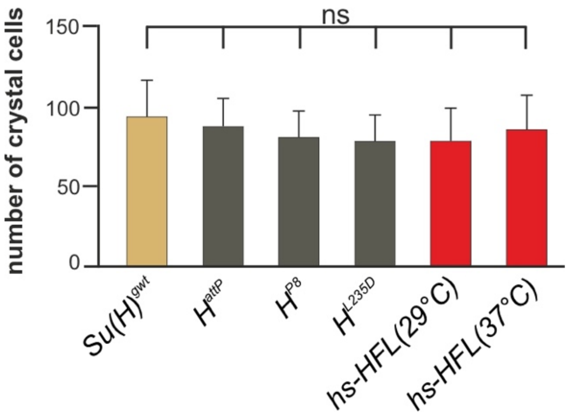

Supplementary Figure 3. Neither Hairless gain- nor loss-of-function affects crystal cell formation

Quantification of melanized crystal cells within the last two segments of homozygous third instar larvae of the given genotype. H^{attP} and H^{P8} are null mutants; H^{L235D} is deficient for Su(H) binding. Hs-HFL was induced either constantly at 29°C, or in a 30 min pulse at 37°C 24 h before analysis (n=50, except for H^{P81} and H^{L235D} with n=20 each). No significant differences were seen relative to control $Su(H)^{gwt}$ (p>0.5, two-tailed Dunnett's test). Error bars represent ± s.d.

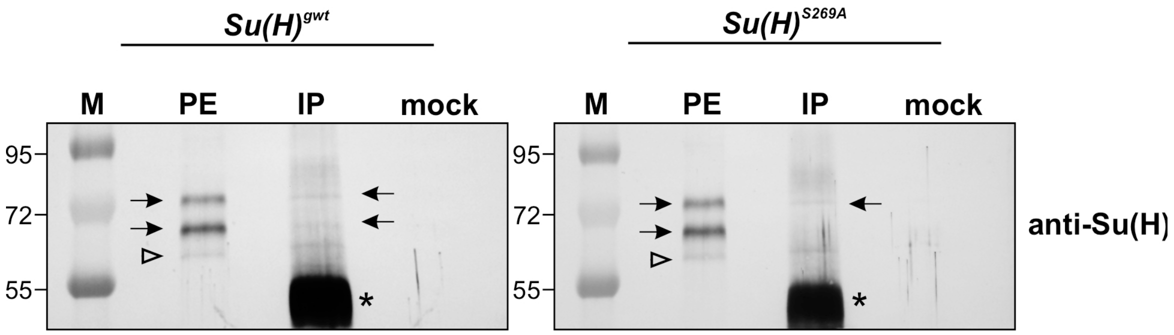

Supplementary Figure 4. Su(H) protein is subjected to phosphorylation in the *Drosophila* embryo

Immunoprecipitation on protein extracts from *Su(H)*^{gwt} (left panel) and *Su(H)*^{S269A} (right panel) embryos, respectively, using phospho-ATM/ATR substrate (S*Q) mAB. The blots were probed with anti-Su(H) antibodies detecting typically two major bands in protein extracts (PE, arrows; 0.7% of total input); smaller bands presumably derive from degradation (arrowheads). Whereas both Su(H) protein species are present in the phospho-fraction of wild type embryonic extracts, only the upper one is robustly detected in the mutant. (* heavy chain Ig signal). M, prestained protein ladder; approximate size in kDA.

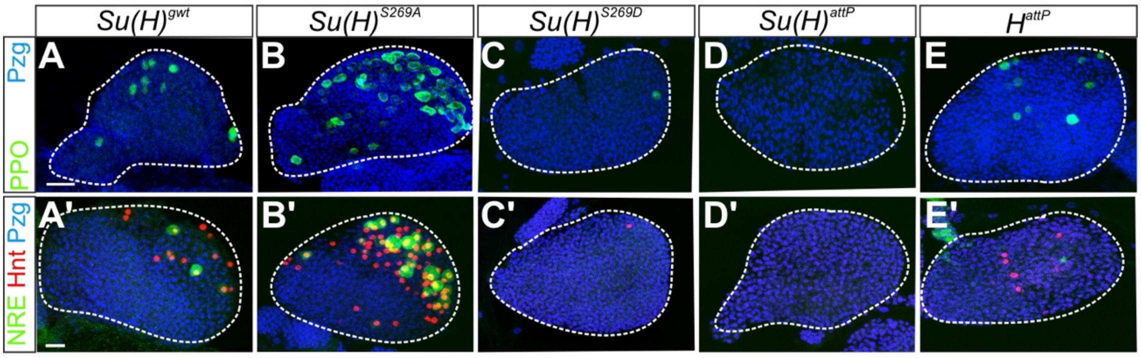

Supplementary Figure 5. PPO and *NRE:EGFP* expression in larval lymph glands

(**A-E**) Primary lobes stained with antibodies against PPO (green) to detect crystal cells and Pzg (blue, nuclear marker) to visualize the outlines. (**A'-E'**) *NRE:EGFP* reporter expression (green) was co-detected with anti-Hnt (red) and anti-Pzg (blue) in primary lobes. (**A,A'**) *Su(H)*^{gwt}, (**B,B'**) *Su(H)*^{S269A}, (**C,C'**) *Su(H)*^{S269D}, (**D,D'**) *Su(H)*^{attP}, (E,E') *H*^{attP}. Scale bar: 20 µm in all panels.

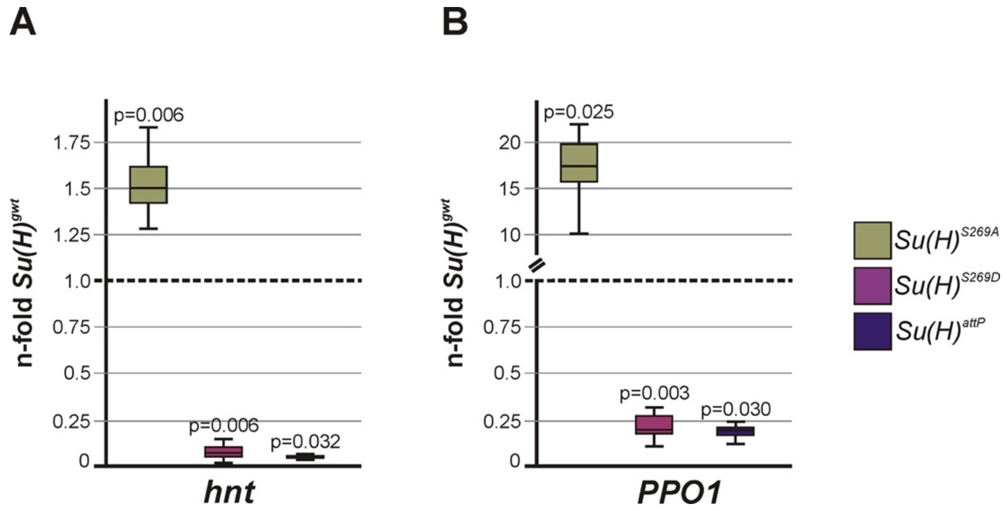

Supplementary Figure 6. qRT-PCR analyses on mRNA from larval lymph glands

qRT-PCR analyses on mRNA from 40 isolated lymph glands each. Both, *hnt* (**A**) and *PPO1* (**B**) transcription is upregulated in $Su(H)^{S269A}$ glands and downregulated in $Su(H)^{S269D}$ and $Su(H)^{attP}$. Four biological and two technical replicates were performed. Amplification efficiencies of reference genes *tbp, cyp33* and *rp49* and tested genes *PPO1* and *hnt* were taken into account for the determination of relative quantities based on REST. Median corresponds to expression ratio; mini-max depicts 95% confidence. All expression ratios shown are significant at the level of p<0.05 using PFRR from REST; p-values are indicated.

Supplementary Table 1. Resources and reagents

**Reagent or Resource**	**Source**	**Identifier**
**Antibodies, Serum and Mounting media**		
Donkey anti-Mouse Cy3	Jackson Immuno-Research Laboratories, (Dianova)	Cat# 715-165-151; RRID: AB_2315777 (1:250)
Donkey anti-Rabbit Cy3	Jackson Immuno-Research Laboratories	Cat# 711-165-152; RRID: AB_2307443 (1:250)
Donkey anti-Rat Cy3	Jackson Immuno-Research Laboratories	Cat# 712-165-153; RRID: AB_2340667 (1:250)
Donkey anti-Mouse FITC	Jackson Immuno-Research Laboratories	Cat# 715-095-151; RRID: AB_2335588 (1:250)
Donkey anti-Guinea Pig Cy5	Jackson Immuno-Research Laboratories	Cat# 706-175-148; RRID: AB_2340462 (1:250)
Goat anti-Horseradish Peroxidase Fluorescein conjugated (anti-HRP-FITC)	Jackson Immuno-Research Laboratories	Cat# 123-095-021; RRID: AB_2314647 (1:250)
Goat anti-Mouse Alkaline Phosphatase	Jackson Immuno-Research Laboratories	Cat# 115-055-003; RRID: AB_2338528 (1:1000)
Goat anti-Rabbit Alkaline Phosphatase	Jackson Immuno-Research Laboratories	Cat# 111-055-003; RRID: AB_2337947 (1:1000)
Goat anti-Mouse Cy3	Jackson Immuno-Research Laboratories	Cat# 115-165-166; RRID: AB_2338692 (1:250)
Goat anti-Rabbit Cy3	Jackson Immuno-Research Laboratories	Cat# 111-165-144; RRID: AB_2338006 (1:250)
Goat anti-Rabbit IgG, HRP-linked	Cell Signaling Technology	Cat# 7074; RRID: AB_2099233 (1:5000)
Goat anti-Rat Cy3	Jackson Immuno-Research Laboratories	Cat# 112-165-167; RRID: AB_2338251 (1:250)

Goat anti-Mouse FITC	Jackson Immuno-Research Laboratories	Cat# 115-095-166; RRID: AB_2338601 (1:250)
Goat anti-Guinea Pig Alexa 647	Jackson Immuno-Research Laboratories	Cat# 106-605-003; RRID: AB_2337446 (1:250)
Guinea Pig anti-Putzig (anti-Pzg)	Own group	Kugler and Nagel, 2007; PMID: 17634285 (1:500)
Horse anti-mouse IgG, HRP-linked	Cell Signaling Technology	Cat# 7076; RRID: AB_330924 (1:5000)
Mouse anti-Cut	Developmental Studies Hybridoma Bank	2B10; RRID: AB_528186 (1:33)
Mouse anti-Flag-M5	Sigma-Aldrich	Cat# F4042: RRID: AB_439686 (1:5000)
Mouse anti-GFP (B2)	Santa Cruz Biotechnology	Cat# sc-9996; RRID: AB_627695 (1:250)
Mouse anti-Hindsight (anti-Hnt)	Developmental Studies Hybridoma Bank	Cat# 1g9; RRID: AB_528278 (1:20)
Mouse anti-PPO	Trenczek group, University of Giessen	12F6; (1:2)
Mouse anti-TBP	Abcam	Cat# ab818; RRID: AB_306337
Mouse anti-betaTubulin	Developmental Studies Hybridoma Bank	E7; RRID: AB_2315513 (1:3000)
Mouse anti-Wingless	Developmental Studies Hybridoma Bank	4D4; RRID: AB_528512 (1:33)
Normal Donkey Serum (NGS)	Jackson Immuno Research Laboratories	Cat# 017-000-121; RRID: AB_2337258
Normal Goat Serum (NGS)	Jackson Immuno Research Laboratories	Cat# 005-000-121; RRID: AB_2336990

Rabbit anti-Su(H)	Santa Cruz Biotechnology	Cat# sc-25761; RRID: AB_672837 (1:500)
Rabbit phospho-ATM/ATR substrate (S*Q) mAB D23H2/D69H5	Cell Signaling Technology	Cat# 9607; RRID: AB_10889739 (1:1000)
Rabbit anti-RBPSuH (D10A4)	Cell Signaling Technology	Cat#5313; RRID: AB_2665555 (1:1000)
Rat anti-Deadpan (anti-Dpn)	Abcam	Cat# ab195173; RRID: AB_2687586 (1:300)
Sheep anti-Digoxigenin-alkaline Phosphatase, Fab fragments	Roche Diagnostic	Cat# 11093274910; RRID: AB_514497 (1:2000)
Sheep anti-mouse IgG, HRP-conjugated	GE-Healthcare	Cat# NA931V; RRID: AB_772210 (1:5000)
Vectashield Mounting Medium	Vector Laboratories	Cat# H-1000; RRID: AB_2336789
**Experimental Models: Organisms and Strains: *Drosophila melanogaster***		
w^{67c23};; *FRT82B* H^{attP}*/TM6B*	D. Maier group, University of Hohenheim	Praxenthaler et al., 2015; PMID: 26448463
H^{P8}*/TM6B*	D. Maier group, University of Hohenheim	Maier et al., 1999; PMID: 10559498
H^{L235D}*/TM6B*	D. Maier group, University of Hohenheim	Praxenthaler et al., 2015; PMID: 26448463
hs H-FL	D. Maier group, University of Hohenheim	Maier et al. 1997; PMID: 9347918
N^{cos479}*/TM6B*	Artavanis-Tsakonas group, Boston, USA	Ramos et al., 1989; PMID: 2555253
$y^1 w^{67c}$; $RBPJ^{wt}$ *FRT40A/CyO-GFP*	Own group	Gahr et al., 2019; PMID: 31615108
$y^1 w^{67c}$; $RBPJ^{S221A}$ *FRT40A/ CyO-GFP*	This work	N/A
$y^1 w^{67c}$; $RBPJ^{S221D}$ *FRT40A/ CyO-GFP*	This work	N/A

y^1w^{67c}; $Su(H)^{attP}$ FRT40A/CyO-GFP	D. Maier group, University of Hohenheim	Praxenthaler et al., 2017; PMID: 28475577
y^1w^{67c}; $Su(H)^{gwt}$ FRT40A	D. Maier group, University of Hohenheim	Praxenthaler et al., 2017; PMID: 28475577
y^1w^{67c}; $Su(H)^{S269A}$ FRT40A	This work	N/A
y^1w^{67c}; $Su(H)^{S269D}$ FRT40A/ CyO-GFP	This work	N/A
y^1 w hs-flipase$^{1.22}$; Ubi-GFP FRT 40A*	Bloomington Stock Center	RRID: BDSC_5189 combined with flipase under heatshock control
y^1 w hs-flipase$^{1.22}$; FRT82B Ubi-GFP*	Bloomington Stock Center	RRID: BDSC_5188 combined with flipase under heatshock control
Vasa-Integrase:<br>*y* w* M{eGFP.vas-int.Dm}ZH-2A*	Basler lab, Zürich	Bischof et al., 2007; PMID: 17360644
OvoD1 FRT40A/Dp(?;2)bw^D/CyO	Bloomington Stock Center	BL2121; RRID: BDSC_2121
NRE:EGFP	Merdes group, Basel	BL30728; RRID:BDSC_30728
**Experimental Models: Cell lines**		
HeLa	ATCC	CCL2
Mouse hybridoma mature T cells	Borggrefe group, University of Giessen	E2-10HA1, Giaimo et al., 2017; PMID: 28027012
Phoenix™	Orbigen	N/A
**Recombinant DNA**		
cDNA clone PPO1 in pOT2 vector (*in situ* probe generation)	Drosophila Genomics Resource Center	GH04080; cDNA Accession AY060617
pcDNA3.1_Flag_mRBPJ_CRr_WT	This work	N/A
pcDNA3.1_Flag_mRBPJ_CRr_S221A	This work	N/A
pcDNA3.1_Flag_mRBPJ_CRr_S221D	This work	N/A
pcDNA3.1_Flag_mRBPJ_CRr-VP16_WT	This work	N/A
pcDNA3.1_Flag_mRBPJ_CRr-VP16_S221A	This work	N/A
pcDNA3.1_Flag_mRBPJ_CRr-VP16_S221D	This work	N/A
pcDNA3.1_hsNICD	Mertens lab, University of Ulm	Close et al., 2019; PMID: 30510140
pGE-attB^{GMR} *$RBPJ^{S221A}$*	This work	N/A

pGE-attB^{GMR} *RBPJ*^{S221D}	This work	N/A
pGE-attB^{GMR} *Su(H)*^{S269A}	This work	N/A
pGE-attB^{GMR} *Su(H)*^{S269D}	This work	N/A
pMYs_IRES_Flag_Blc	This work	N/A
pMYs_Flag_mRBPJ_CRr_WT_IRES_Blc	This work	N/A
pMYs_Flag_mRBPJ_CRr_S221A_IRES_Blc	This work	N/A
pMYs_Flag_mRBPJ_CRr_S221D_IRES_Blc	This work	N/A
12xCSL-RE (pGa981/6)	Honjo lab, Kyoto University	Minoguchi et al., 1997; PMID: 9111338
**Critical Commercial Assays and chemicals**		
Blasticidin	Gibco	Cat# A1113903
Bio-Rad Protein Assay - Dye Reagent Concentrate	Bio-Rad	Cat# 500-0006
Chloroquine	Sigma-Aldrich	Cat# C6628-100G
cOmplete^{TM} Ultra Tablets Protease Inhibitor Cocktail	Roche	Cat# 05892791001
Lipofectamine 2000	Thermo Fisher	Cat# 11668019
Luciferase Assay System	Promega	Cat# E1501
PhosSTOP^{TM}	Roche	Cat# 04906837001
Polybrene - transfection reagent	Sigma-Aldrich	Cat # TR-1003_5G
poly(dI-dC) sodium salt	Merck	Cat# P4929-25UN
Profectin©Mammalian Transfection System	Promega	Cat# E1200
Protein A-Agarose	Roche	Cat# 11134515001
Q5®Site-Directed Mutagenesis Kit	New England Biolabs	Cat# E0554
QuickChange II XL Site_directed Mutagenesis Kit	Agilent	Cat# 200521-5
SuperSignal^{TM} West Pico PLUS Chemiluminescent Substrate	Thermo Fisher Scientific	Cat# 34580
TNT-Assay (T7)	Promega	Cat# L4610
**Oligonucleotides for EMSAs and mutagenesis (5'-3')**		
FO233_F: RBPJ binding sites underlined: CCTGGAACTATTTTCCCACGGTGCCCTTCCGCCCATTTTCCCACGAGTCG	Biomers	N/A
FO233_R: RBPJ binding sites underlined: CTCGCGACTCGTGGGAAAATGGGCGGAAGGGCACCGTGGGAAAATAGTTC	Biomers	N/A
dRBPJ S221A_mut_UP: CAATCGCCTTCGTGCACAGACAGTTAG	Microsynth	N/A
dRBPJ S221A_mut_LP: AACAGTGCCACCTTCGTTC	Microsynth	N/A
dRBPJ S221D_mut_UP: GTTAGTACTAGGTACCTGCATGTAGAAG	Microsynth	N/A
dRBPJ S221D_mut_LP: TGTCTGGTCCCGAAGGCGATTGAACAG	Microsynth	N/A

mRBPJ S221A_UP: GCACTGTTCAATCGCCTTCGGG CGCAGACAGTTAGTACCAGG	Microsynth	N/A
mRBPJ S221A_LP: CCTGGTACTAACTGTCTGCGCC CGAAGGCGATTGAACAGTGC	Microsynth	N/A
mRBPJ S221D_UP: GCACTGTTCAATCGCCTTCGGG ACCAGACAGTTAGTACCAGG	Microsynth	N/A
mRBPJ S221D_LP: CCTGGTACTAACTGTCTGGTCC CGAAGGCGATTGAACAGTGC	Microsynth	N/A
**Software and Algorithms**		
Figure assembly	CorelDRAW® Graphics Suits Version 9	RRID:SCR_ 014235
Data processing and analyzing	Fiji (Image J)	RRID:SCR_ 003070
Picture processing and analyzing	Corel Photo Paint® Version 9.337	N/A
LaserSharp 2000 software for MRC1024	Bio-Rad	N/A
Data analysis and graphing	Origin Pro	RRID:SCR_ 014212
Statistical evaluation	ANOVA	
Statistical significance	Dunnett’s test	Dunnett, 1955; PMID: 14368526
Statistical significance	Tukeys-Kramer	Tukey, 1949; PMID: 18151955
MIC PCR version 2.10.0	bms/Biozym	Cat# 68MIC-HRM
**Materials, oligonucleotides and equipment for qRT-PCR**		
Absolute QPCR ROX Mix (for qPCR in mature T-cells)	Thermo Scientific	Cat# AB1138B
Blue S'Green qPCR Kit (for qPCR in *Drosophila* tissue)	Biozym	Cat# 331416
Dynabeads™ mRNA DIRECT™ Micro Purification Kit	Invitrogen, Thermo Fisher Scientific	Cat# 61021
Dynabeads™ mRNA DIRECT™ Purification Kit	Invitrogen, Thermo Fisher Scientific	Cat# 61011
DNaseI	New England Biolabs	Cat# M0303
M-MuLV reverse transcriptase	New England Biolabs	Cat# M0253S
qScriber™ cDNA Synthesis Kit	highQu	Cat# RTK0104
TRIzol	Ambion	Cat# 15596018
Mic Magnetic Induction Cycler (for qPCR in *Drosophila* tissue)	bio mol. systems/ Biozym	Cat# 68MIC-2
StepOne Plus ™ - Real Time PCR System (for qPCR in mature T-cells)	AB / Thermo Fisher Scientific	Cat# 4376600
Primers for fly qRT-PCR:		

cyp33_UP: CTCTGCGGACGCACAATTC	Microsynth	PP14577 DRSC Fly-PrimerBank
cyp33_LP: TGCAACCAGTCGTCATCTGC	Microsynth	PP14577 DRSC Fly-PrimerBank
dpn_UP: CCGGCTCGTCATACCAAACTG	Microsynth	PP17352 DRSC Fly-PrimerBank
dpn_LP: CGTCTTGAACTTCTGGACAACG	Microsynth	PP17352 DRSC Fly-PrimerBank
E(spl)m3_UP: CAACAAGTGTCTGGACGATCTC	Microsynth	PP7204 DRSC Fly-PrimerBank
E(spl)m3_LP: ATGTGATCCACGGTCAACTC	Microsynth	PP7204 DRSC Fly-PrimerBank
E(spl)m8_UP: ATGGAATACACCACCAAGACC	Microsynth	PP10017 DRSC Fly-PrimerBank
E(spl)m8_LP: GGCGACAAGTGTTTTCAGGTT	Microsynth	PP10017 DRSC Fly-PrimerBank
peb/hnt_UP: GAGCGGCCATTCCAGTGTGA	Microsynth	N/A
peb/hnt_LP: TTGTTGTTGGCGCTGGTCGG	Microsynth	N/A
proPo_A1_1_UP: TTGGAACTGCCCGATTCCTTC	Microsynth	PP22066 DRSC Fly-PrimerBank
proPo_A1_1_LP: TTCAGATCCACGTCCTTAGAGAA	Microsynth	PP22066 DRSC Fly-PrimerBank
rp49_UP: AGCATACAGGCCCAAGATCG	Microsynth	PD41810 DRSC Fly-PrimerBank
rp49_LP: TGTTGTCGATACCCTTGGGC	Microsynth	PD41810 DRSC Fly-PrimerBank
tbp_UP: TAAGCCCCAACTTCTCGATTCC	Microsynth	PP1556 DRSC Fly-PrimerBank
tbp_LP: GCCAAGAGACCTGATCCC	Microsynth	PP1556 DRSC Fly-PrimerBank
Primers for mouse qRT-PCR:		
Hes1_UP: TGCCAGCTGATATAATGGAGAA	Microsynth	N/A
Hes1_LP: CCATGATAGGCCTTTGATGACTTT	Microsynth	N/A
Hprt_UP: GGAGCGGTAGCACCTCCT	Microsynth	N/A
Hprt_LP: AACCTGGTTCATCATCGCTAA	Microsynth	N/A
Lgmn_UP: GAATTCCCACGGTTCTGC	Microsynth	N/A
Lgmn_LP: AGCACCAGGCTGAGAAGC	Microsynth	N/A
Notch1_UP: TGACCTGCTCACTCTCACAGA	Microsynth	N/A
Notch1_LP: TCAGCCTGCTGACATGATTT	Microsynth	N/A
Tbp_UP: GGGGAGCTGTGATGTGAAGT	Microsynth	N/A
Tbp_LP: CCAGGAAATAATTCTGGCTCAT	Microsynth	N/A
